# Protocol for cellular RNA G-quadruplex profiling using G4RP.v2

**DOI:** 10.1016/j.xpro.2024.103480

**Published:** 2024-12-10

**Authors:** Jérémie Mitteaux, David Monchaud

**Affiliations:** 1 Institut de Chimie Moléculaire de l’Université de Bourgogne (ICMUB), CNRS UMR 6302, 9 Avenue Alain Savary, 21078 Dijon, France

**Keywords:** Biophysics, Genetics, Molecular Biology, Chemistry

## Abstract

The isolation of G-quadruplexes (G4s) from human cells using specific molecular tools constitutes an invaluable step forward in uncovering the biology of these higher-order DNA and RNA structures. Here, we present an improved version of the G4-RNA precipitation (G4RP) protocol developed to identify RNA G4s from human cancer cells. We describe steps for cell treatment and lysis, chemoprecipitation of G4s using TASQ tools, go/no-go steps, and quantitative reverse-transcription PCR (RT-qPCR) quantification and analysis.

For complete details on the use and execution of this protocol, please refer to Mitteaux et al.^1^

## Highlights

• Step-by-step procedure for the chemoprecipitation of G-quadruplex (G4) RNA
• Introduction of in-column purification of precipitated G4 RNAs
• Example of quantification for purified *NRAS* and *VEGFA* mRNA G4s by RT-qPCR
• Alternative and go/no-go steps for users’ convenience and preference

## Abstract (editor highlights)

Publisher’s note: Undertaking any experimental protocol requires adherence to local institutional guidelines for laboratory safety and ethics.

## Graphical abstract

**Figure:**
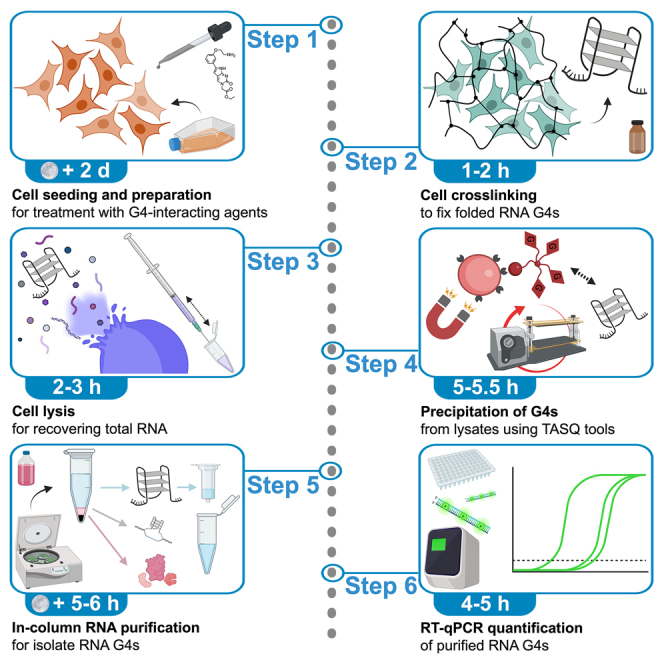

## Before you begin

G-quadruplexes (G4s)^2^ fold from guanine (G)-rich DNA^3^^,^^4^^,^^5^ and RNA^6^^,^^7^^,^^8^^,^^9^ sequences, which are highly abundant (> 1 million sequences) in the genome^10^ and transcriptome^11^ of human cells. The knowledge about G4 prevalence was acquired thanks to different techniques, either *in silico*^12^ or *in vitro*,^13^ the latter relying, for instance, either on polymerase stop assays (e.g., G4-seq for DNA G4,^14^ rG4-seq for RNA G4)^15^ or on the precipitation of G4s using *ad hoc* molecular tools (either antibodies,^16^^,^^17^^,^^18^^,^^19^^,^^20^ proteins^21^ or small molecules).^22^^,^^23^^,^^24^^,^^25^ Our contribution to this research effort was the development of the G4-RNA-specific precipitation (G4RP) protocol developed to identify RNA G4s that fold in *in vivo* conditions in human cancer cells. Two versions of this protocol were developed, depending on whether the analysis is targeted (G4RP-RT-qPCR)^26^ or transcriptome-wide (G4RP-seq).^23^^,^^27^

We report herein on a novel version of this protocol, named G4RP.v2, the key step of which is the specific precipitation of G4s using TASQs (for template-assembled synthetic G-quartets),^28^^,^^29^^,^^30^ a series of biomimetic ligands that display both high affinity and exquisite selectivity for G4s. The responsiveness of the G4RP.v2 protocol makes it suited to finely monitor the modulation of G4 landscapes in cells by chemical effectors (either G4 stabilizers^31^ or destabilizers)^32^; its reliability allows for asserting the G4 folding of PQS in a functional cellular context, often referred to as *in vivo*-like conditions given that cellular G4s are fixed prior to cell lysis and isolation (further detailed below); finally, its accuracy makes it ideally positioned to be used for the quantification of G4-containing genes expression in cancer cells.

The protocol fully described below focuses on the application of G4RP.v2 for the accurate quantification by RT-qPCR of two G4-containing mRNAs, i.e., *NRAS*^33^ and *VEGFA*,^34^ as representative examples. To demonstrate the scope of application of G4RP.v2, these investigations were performed in human breast cancer cells, i.e., MCF7, treated (or not) with two G4 modulators: the G4 stabilizer BRACO-19^35^ or the G4 destabilizer PhpC.^36^ These investigations serve multiple purposes, mainly aiming at validating *i*- the G4 targets of these chemicals, for mechanistic investigations and/or interpretations, and *ii*- the G4 folding of these PQSs in a functional cellular context (*in vivo*) by both positive and negative regulation using *ad hoc* chemical effectors, an approach that thus fully abides by the definition of chemical biology.

Our results show that PhpC treatment induces a significant decrease in the quantity (RT-qPCR fold change) of precipitated *NRAS* (using three TASQs as molecular baits, *i.e*., BioTASQ,^23^^,^^26^ BioCyTASQ^37^^,^^38^ or the biotin-clicked ^az^MultiTASQ^39^) and *VEGFA* (only with BioCyTASQ or biotin-clicked ^az^MultiTASQ) (see the resource availability section),^1^ while the BRACO-19 treatment showed a global increase in the quantity of precipitated mRNA but in a more erratic manner, especially with *VEGFA* mRNA, indicating that the implemented conditions could be further optimized for improving the quality of the output (see the resource availability section).

Three TASQs were selected for these investigations, i.e., BioTASQ, the first prototype of biotinylated TASQ used in the original G4RP protocol;^23^^,^^26^^,^^27^^,^^40^ *ii.* BioCyTASQ, a structurally simplified analog of BioTASQ,^37^ which was also used in a G4RP-like protocol dedicated to DNA G4s (G4DP-seq)^24^; and the clickable ^az^MultiTASQ,^39^ used here after biotinylation by click chemistry, which was embedded in the development of the G4RP.v2 protocol.^1^ These three TASQs were used to demonstrate the generality of the protocol but only one TASQ is necessary to perform G4RP.v2 experiments.

The G4RP.v2 protocol could be performed in a total of 7 consecutive days and is divided into three main steps (with possible pauses, as indicated below): *i*- the preparation phase (1–2 days, plus the time required for the preparation of cells); *ii*- the cell culture and treatment (1 night plus 2 days), and *iii*- the implementation of the G4RP.v2 protocol *per se* (2 days). Each of these main steps, along with the many constitutive tasks and sub-tasks, will be accurately detailed below.

### Benchtops and materials preparation

**Timing: 10 min**

1. Clean benchtops (e.g., classical benchtop, benchtop under a fume hood) and materials (e.g., pipette, racks) with the RNase decontaminant RNaseZap.

### Cells preparation

**Timing: variable (depending on cell lines)**

2. Thaw MCF7 cells and plate them in a culture flask.
  ***Note:*** The choice of the cell culture plastic and size depends on habits and stock cell density.
  **CRITICAL:** Cells have to be manipulated under a standard biosafety cabinet to keep sterile condition.
  a. Add supplemented DMEM culture medium.
  b. Let cells grow overnight in a standard cell culture incubator in appropriate conditions (for MCF7: 37°C, 5% CO_2_).
  c. The next day, replace the culture medium with fresh medium.
    **CRITICAL:** The culture medium replacement will allow for removing the DMSO present in the stock culture medium, which has a long-term cytotoxic effect.
3. Let the cells grow and perform cells passages when needed.
  a. Remove the cell culture medium.
  b. Wash cells with sterile 1X PBS.
  c. Add trypsin to detach cells.
  d. Incubate at 37°C for 3–5 min (in the incubator).
  e. Check under the microscope that the cells have been completely detached from the flask; if not, clap the flask gently over a paillasse and check again.
  f. Add an equivalent of supplemented DMEM culture medium to neutralize trypsin.
  g. Divide cells at a density of choice in a new flask with fresh culture medium.
  h. Let them grow into the incubator.

***Note:*** MCF7 cells have a doubling time of approximately 24 h. Three passages per week are reasonable. For having enough cells for performing G4RP.v2, cells can be cultured in T175 cm^2^ flasks.

**CRITICAL:** Maintain cells about one week in culture before starting the G4RP.v2 protocol to let them recover from the freezing/thawing cycles.

### Denaturing agarose gel preparation

**Timing: 40 min**

4. In case you are used to prepare agarose gel manually, you can prepare one and then store it.

***Note:*** See the materials and equipment section for a detailed preparation of denaturing agarose gel.

**Pause point:** The gel can be easily stored for 3–4 days at 4°C if immersed in the running buffer (i.e., TBE buffer 1X).

### G4-interacting agents and molecular TASQ tools preparation

**Timing: 1–2 day(s)**

5. Prepare BRACO-19 solution at 2 mM, and PhpC solution at 20 mM.

**Figure 1:**
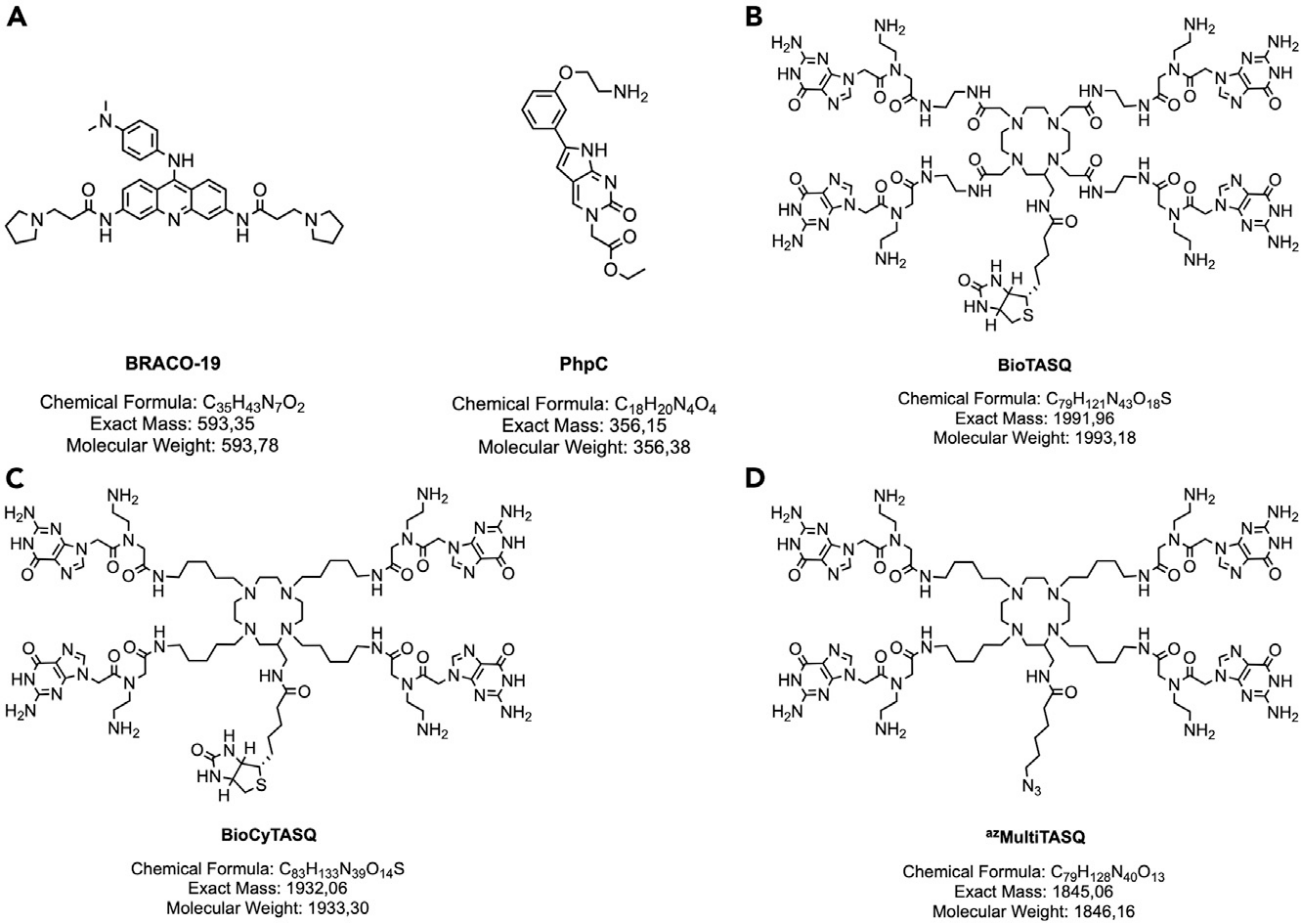
Structure of the G4-interacting agents and the G4-specific TASQ tools used Several compounds were used in the G4RP.v2 protocol: (A) BRACO-19 (left) and PhpC (right), (B) BioTASQ, (C) BioCyTASQ and (D) ^az^MultiTASQ. See also Tables 1 and 2.

***Note:*** See the materials and equipment section for details about the preparation of these small molecules, and Figure 1 for structure and chemical information.

6. Prepare BioCyTASQ, BioTASQ and/or biotin-clicked ^az^MultiTASQ solution at 1 mM, and biotin-PEG(4)-DBCO solution for coupling with ^az^MultiTASQ.

**Table 1:** Molar weight and exact mass of TASQs

Molecular TASQ tools	W/ TFA	W/O TFA
**Molar weight (g/mol)**		

^az^MultiTASQ	2,302.24	1,846.16
BioCyTASQ	2,389.38	1,933.30
BioTASQ	2,449.26	1,993.18
Biotin-clicked ^az^MultiTASQ	N/A	2,596.08

**Exact mass (Da)**		

^az^MultiTASQ	N/A	1,845.06
BioCyTASQ	N/A	1,932.06
BioTASQ	N/A	1,991.96
Biotin-clicked ^az^MultiTASQ	N/A	2,594.40

The molar weight and exact mass of molecular G4-specific TASQ vary through their solubilization processes (i.e., TASQ powder contain TFA molecules).

**Table 2:** Exact mass of the different forms of biotin-clicked ^az^MultiTASQ

Name	Exact mass (Da)	m/z ratio				
		z = +1	z = +2	z = +3	z = +4	z = +5
^az^MultiTASQ	1,845.06	predicted values for the [M + H]^z^ ion				
		1,846.06	923.03	615.35	461.52	369.21
		measured values				
		N/A	923.70	616.30	462.20	N/A
Biotin-PEG(4)-DBCO	749.35	predicted values for the [M + H]^z^ ion				
		750.35	375.18	250.12	187.59	150.07
		measured values				
		750.40	375.70	N/A	N/A	N/A
Biotin-clicked ^az^MultiTASQ	2,594.40	predicted values for the [M + H]^z^ ion				
		2,595.40	1,297.7	865.13	648.85	519.08
		measured values				
		N/A	1,297.80	866.70	650.00	520.30

**Figure 2:**
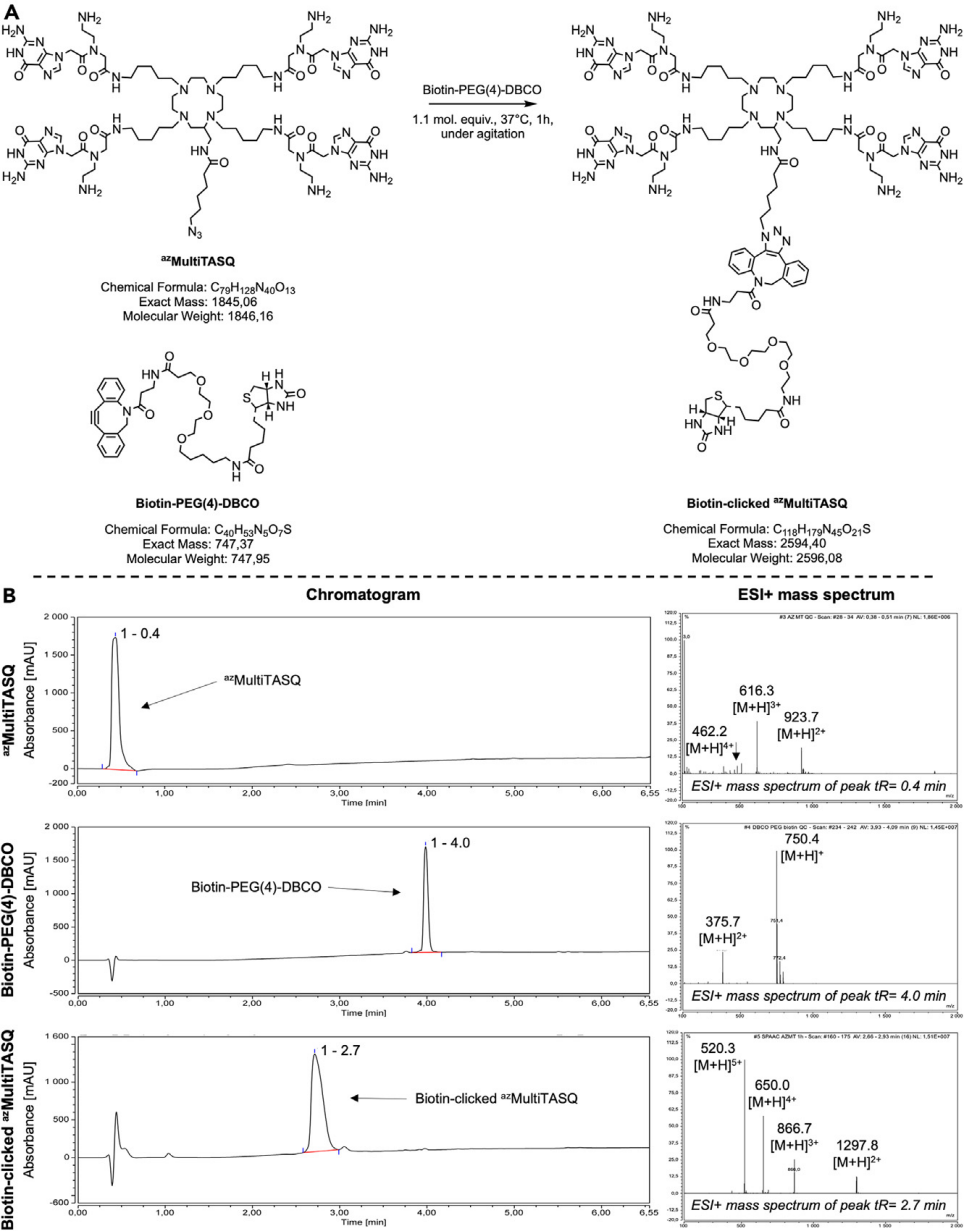
Click chemistry reaction to generate biotin-clicked ^az^MultiTASQ and its monitoring by HPLC-MS This biotin-clicked molecular tool is obtained after a (A) click reaction (SPAAC) between the ^az^MultiTASQ and biotin-PEG(4)-DBCO (37°C, 1 h, under agitation) and this reaction can be monitored by (B) HPLC-MS (acetonitrile increasing gradient; flow rate = 0.500 mL/min) in analyzing the m/z ratios of ions generated (see Tables 1 and 2).

***Note:*** see the materials and equipment section for details about the preparation of these small molecules, Figure 1 and Table 1 for the structure and chemical information required for the preparation of the three TASQs, and Figure 2 and Table 2 for the condition and characterization of the click reaction to generate the biotin-clicked ^az^MultiTASQ.

The preparation of the biotin-clicked ^az^MultiTASQ can be monitored by HPLC-MS analysis (see Figure 2). The m/z ratio corresponds to the exact mass of this [M + H]^z^ ion divided by its charge number. Experimental (“measured”) values were obtained. “N/A” means no peak has been strongly detected.

**CRITICAL:** An HPLC-MS analysis is mandatory to ascertain the efficiency of the click reaction.

***Note:*** Count *ca.* 4 molecules of Trifluoroacetic acid (TFA) per each TASQ molecule, which correspond to a molar weight increment/decrement of 456.08 g/mol. See Figures 1 and 2 for more chemical information about these TASQs.

### Programs preparation

**Timing: 30 min**

7. Select and/or prepare the programs for the Reverse Transcription (RT) and real-time PCR (qPCR) on the selected thermocycler (in our case, Agilent Mx3005P).

**Table 3:** Reverse transcription and real-time PCR programs

Step	Time (min:sec)	Temperature (°C)	Number of cycles
**Reverse transcription (RT) program (75:00)**			

Initial incubation	05:00	25	1
Elongation	45:00	55	1
Polymerase denaturation	15:00	70	1
Final incubation	10:00	25	1

**Real-time PCR (qPCR) program: Amplification part (130:00)**			

Initial denaturation	10:00	95	1
Denaturation	00:30	95	60
Hybridization	00:45	60	
Elongation	00:45	72	

**Real-time PCR (qPCR) program: Dissociation part (02:30)**			

Denaturation	01:30	95	1
Hybridization	00:30	55	1
Denaturation	00:30	95	1

**Figure 3:**
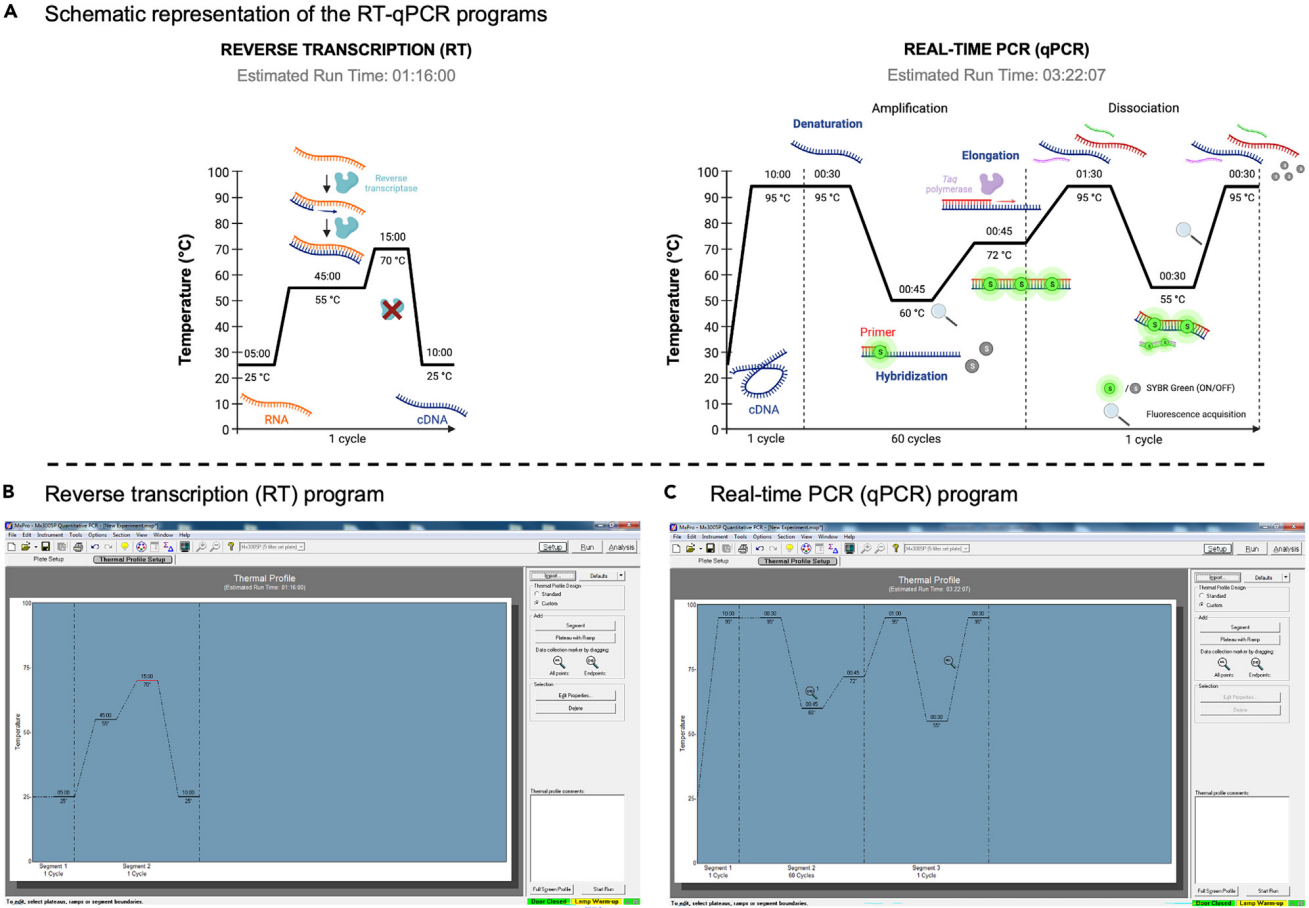
Programs for the Reverse Transcription and qPCR steps in the G4RP.v2 protocol (A) The G4RP.v2 protocol ends with the Reverse Transcription (RT) and real-time PCR (qPCR) reactions in order to measure the quantity of specific mRNAs. (B and C) Two procedures were created to automate these two reactions. Times in hour:min:sec (or just min:sec). (A) was created with BioRender. See also Table 3.

***Note:*** see the materials and equipment section, Figure 3 and Table 3 for details about the preparation of these programs.

The RT reaction has a duration of 75 min and comprises an initial incubation followed by the RT where RNA is reverse transcribed in cDNA, the denaturation of the reverse transcriptase (the SuperScript III) and then a final incubation. The qPCR reaction is divided in two distinct steps: the qPCR amplification and the dissociation part. The qPCR amplification has a duration of 130 min and, after a first initial denaturation, allows for an amplification of cDNA by 60 (can be reduced to 35) cycles of denaturation, hybridization and elongation. The dissociation part which comes after has a duration of 2:30 min and allows for the evaluation the homogeneity of amplicons and then, the specificity of the qPCR. See also Figure 3.

### qPCR primers couples preparation

**Timing: 30 min**

8. Prepare the qPCR primers couples.

***Note:*** See the materials and equipment section for details about the preparation of these qPCR primers couples.

**CRITICAL:** The use of an UltraPure DNase/RNase-free distilled water is essential for the RT-qPCR to avoid contamination and/or degradation of purified samples.

### Reagents preparation

**Timing: 1–2 days**

9. Prepare the following buffers and solutions: Biotin (1 mM, 10 mM), BRACO-19 (2 mM), DEPC-H_2_O (0.1% v/v), EDTA buffer (0.5 M, pH 8.0), EGTA buffer (0.5 M, pH 8.0), fixing buffer (5X), G4RP buffer (pH 7.4), glycine (1 M), HCl (1 M), HEPES KOH buffer (1 M, pH 7.5), KCl (1 M), KOH (1 M), NaCl (1 M), NaOH (1 M), PhpC (20 mM), SDS (20% w/v), supplemented DMEM culture medium, TBE buffer (10X, pH 8.3), TBE buffer (1X).

***Note:*** See the materials and equipment section for details about the preparation of these reagents.

**CRITICAL:** The use of RNase-free water (e.g., ddH_2_O or DEPC-treated water) is critical to both keep the integrity of RNA and maximize their recovery after precipitation and purification.

**CRITICAL:** Prepare all reagents (or at least the final buffers and solutions) in a sterile environment (e.g., under a standard biosafety cabinet, with 0.2-μm filtration, etc.).

### RNA Clean & Concentrator-5 kit preparation

**Timing: 10 min**

10. Prepare DNase I and RNA Wash Buffer solutions from the RNA Clean & Concentrator-5 kit.

***Note:*** See the materials and equipment section for details about the preparation of these RNA Clean & Concentrator-5 kit solutions.

**CRITICAL:** The use of RNase-free water (e.g., ddH_2_O or DEPC-treated water) is critical to both keep the integrity of RNA and maximize their recovery after precipitation and purification.

## Key resources table

**Table:** 

REAGENT or RESOURCE	SOURCE	IDENTIFIER
**Chemicals, peptides, and recombinant proteins**		

Agarose (SeaKem LE)	Fisher Scientific	Cat# LON50004; CAS: 9012-36-6
^az^MultiTASQ or azidoMultiTASQ (MW = 1,846.16 g/mol)^a^	Laboratory of David Monchaud (Dijon, France)^b^	N/A
BioCyTASQ (MW = 1,933.30 g/mol)^a^	Sigma-Aldrich	Cat# SCT246
BioTASQ (MW = 1,993.18 g/mol)^a^	Laboratory of David Monchaud (Dijon, France)^b^	N/A
Biotin (MW = 244.31 g/mol)	Sigma-Aldrich	Cat# B4501; CAS: 58-85-5
Biotin-PEG(4)-DBCO (MW = 749.92 g/mol)	Iris Biotech	Cat# RL-2520; CAS: 1255942-07-4
Bleach or sodium hypochlorite (2.6% w/v)	UGAP	Cat# 619; CAS: 7681-52-9
BRACO-19 (hydrochloride) (≥96%; MW = 593.76 g/mol)	Sigma-Aldrich	Cat# SML0560; CAS: 1177798-88-7
Chloroform (≥99.9%)	Dutscher	Cat# 438603; CAS: 67-66-3
DEPC or Diethylpyrocarbonate (96%)	Sigma-Aldrich	Cat# D5758; CAS: 1609-47-8
DMSO or dimethyl sulfoxide (≥99.9%)	Sigma-Aldrich	Cat# D8418; CAS: 67-68-5
DTT or DL-dithiothreitol (≥98%)	Sigma-Aldrich	Cat# D0632; CAS: 3483-12-3
EDTA or ethylenediamine tetraacetic acid (99%; MW = 292.25 g/mol)	VWR	Cat# TS11843; CAS: 60-00-4
EGTA or ethylene glycol-bis(2-aminoethylether)-*N,N,N′,N′*-tetraacetic acid (≥97%; MW = 380.35 g/mol)	Sigma-Aldrich	Cat# E3889; CAS: 67-42-5
Ethanol absolute (≥99.8%)	VWR	Cat# 15338; CAS: 64-17-5
Formaldehyde solution (16% w/v, in PBS)	Fisher Scientific	Cat# 28908; CAS: 50-00-0
Glycine (≥99%; MW = 75.07 g/mol)	Sigma-Aldrich	Cat# G8898 CAS: 56-40-6
HCl or hydrogen chloride (37% m/m, *ca.* 11.97 M; MW = 36.46 g/mol)	Fisher Scientific	Cat# H/1150/PB17; CAS: 7647-01-0
HEPES or 4-(2-hydroxyethyl)piperazine-1-ethanesulfonic acid, *N*-(2-hydroxyethyl)piperazine-*N′*-(2-ethanesulfonic acid) (99%; MW = 238.30 g/mol)	Fisher Scientific	Cat# AC172571000; CAS: 7365-45-9
KCl or potassium chloride (99.0%–100.5%; MW = 74.55 g/mol)	Sigma-Aldrich	Cat# P3911; CAS: 7447-40-7
KOH or potassium hydroxide (≥85%; MW = 56.105 g/mol)	Fisher Scientific	Cat# 306031; CAS: 1310-58-3
NaCl or sodium chloride (99.5%–100.5%; MW = 58.44 g/mol)	VWR	Cat# 27810; CAS: 7647-14-5
NaOH or sodium hydroxide (100%; MW = 39.997 g/mol)	Fisher Scientific	Cat# S/4920/60; CAS: 1310-73-2
Orthoboric acid (99.8%–100.5%; MW = 61.83 g/mol)	VWR	Cat# 20185; CAS: 10043-35-3
PBS or Dulbecco’s phosphate-buffered saline (10X)	Dutscher	Cat# X0515
PBS or Dulbecco’s phosphate-buffered saline (1X)	Dutscher	Cat# L0615
PhpC or phenylpyrrolocytosine (MW = 356.38 g/mol)	Laboratory of Robert H. E. Hudson (London, Canada)^b^	N/A
SDS or sodium dodecyl sulfate (≥98.5%; MW = 288.38 g/mol)	Sigma-Aldrich	Cat# L3771; CAS: 151-21-3
Tergitol (type NP-40, 70% w/v)	Sigma-Aldrich	Cat# NP40S
TFA or trifluoroacetic acid (≥99%; MW = 114.02 g/mol)	Fisher Scientific	Cat# T/3258; CAS: 76-05-1
Trizma base (MW = 121.14 g/mol)	Sigma-Aldrich	Cat# 933632; CAS: 77-86-1
UltraPure DNase/RNase-free distilled water	Fisher Scientific	Cat# 10977035

**Critical commercial assays**		

RNA Clean & Concentrator-5 kit (supplied with DNase I)	Zymo Research	Cat# R1013
SuperScript III reverse transcriptase kit (which contains SuperScript III reverse transcriptase 200 U/μL, 5X first-strand buffer, and DTT 0.1 M)	Fisher Scientific	Cat# 18080044

**Deposited data**		

Analyzed data and their representation	Mitteaux et al.^1^	https://doi.org/10.1039/d3cc05155b
Exploratory data	These^41^	https://theses.fr/s227390
Raw and analyzed data	FigShare^42^	https://figshare.com/articles/dataset/G4RP_v2_raw_data_for_NRAS_VEGFA_xlsx/25957162

**Experimental models: Cell lines**		

*H. sapiens*: MCF7 cells	ECACC	Cat# 86012803; RRID: CVCL_0031

**Oligonucleotides**		

*NRAS* forward primer (23 bases): d[^5’^ATGACTGAGTACAAACTGGTGGT^3’^]	Yang et al.^2^	N/A
*NRAS* reverse primer (23 bases): d[^5’^CATGTATTGGTCTCTCATGGCAC^3’^]	Yang et al.^2^	N/A
*VEGFA* forward primer (20 bases): d[^5’^CCTTGCCTTGCTGCTCTACC^3’^]	Yang et al.^2^	N/A
*VEGFA* reverse primer (20 bases): d[^5’^AGATGTCCACCAGGGTCTCG^3’^]	Yang et al.^2^	N/A

**Software and algorithms**		

Cary WinUV software (for the UV-vis spectrophotometer)	Agilent	N/A
Data treatment software (Excel/OriginPro, version 2018)	Microsoft Corp./OriginLab Corp.	N/A
MxPro QPCR software (for the real-time PCR system and thermocycler)	Agilent	N/A

**Other**		

Bottle filter, 0.22 μm (Stericup quick release vacuum filtration system)	Sigma-Aldrich	Cat# S2GPU10RE
Cell counting chamber (Neubauer) (optional)	Fisher Scientific	Cat# 717820
Cell scraper (optional)	Fisher Scientific	Cat# 08-100-241
dNTP mix (10 μM)	Fisher Scientific	Cat# R0191
DMEM or Dulbecco’s modified Eagle’s medium (high glucose, w/ L-glutamine, w/ sodium pyruvate)	Dutscher	Cat# L0104
Douncer (optional)	Wheaton	Cat# 357544
Electrophoresis system (mini horizontal electrophoresis system for 105 × 83 mm and 50 × 83 mm)	Dutscher	Cat# MT108
FBS or fetal bovine serum	Dutscher	Cat# S1810
Gel imager (ChemiDoc MP imaging system)	Bio-Rad	Cat# 12003154
Hypodermic needle, G27 (0.40 × 40 mm, gray)	Fisher Scientific	Cat# 9186182
i*Taq* Universal SYBR Green supermix (2X)	Bio-Rad	Cat# 1725121
Lyophilizer	Christ	Alpha 2-4 LD Plus
Magnetic rack (Magna GrIP rack)	Sigma-Aldrich	Cat# 20-400
PCR plate, 96-wells (+ optical strip caps)	Agilent	Cat# 401334 (Cat# 401425)
Pen-Strep or penicillin-streptomycin (Pen: 10,000 U/mL; Strep: 10,000 μg/mL)	Fisher Scientific	Cat# 151-40-122
Random hexamers (50 μM)	Fisher Scientific	Cat# N8080127
Real-time PCR system and thermocycler	Agilent	Mx3005P
RiboRuler high range RNA ladder, ready-to-use (which contains RiboRuler high range RNA ladder and 2X loading dye solution)	Thermo Fisher Scientific	Cat# SM1823
RNaseZap, RNase decontamination solution	Fisher Scientific	Cat# AM9780
RNaseOUT, recombinant ribonuclease inhibitor (100 mM, 40 U/μL)	Fisher Scientific	Cat# 10777019
Standard biosafety cabinet (MSC-Advantage 1.2)	Thermo Fisher Scientific	Cat# 51025411
Standard cell culture CO_2_ incubator (BB 15)	Thermo Fisher Scientific	Cat# 51023121
Standard fume hood	ILM Agencements	N/A
Streptavidin MagneSphere paramagnetic particles (beads) (1 mg/mL)	Promega	Cat# Z5481
SYBR Gold nucleic acid gel stain (10,000X)	Fisher Scientific	Cat# S11494
Syringe, 1 mL (Injekt-F Solo 1 mL)	Fisher Scientific	Cat# 9166017V
Syringe, 50 mL (Omniflix Solo Luer-Lock 3-piece)	Fisher Scientific	Cat# 4617509F
Syringe filter, 0.2 μm (Minisart NML)	Sartorius	Cat# 16534-K
TRIzol	Fisher Scientific	Cat# 15596026
Trypsin (0.25% w/v, in PBS, w/o calcium, w/o magnesium, w/o phenol red)	Dutscher	Cat# L0910
UV-vis spectrophotometer	Agilent	Varian Cary 50 Probe

a Please see the materials and equipment section, Table 1, and Figures 1 and 2 for the precise molar weight of ^az^MultiTASQ, BioCyTASQ and BioTASQ and derivatives.

b Please see the resource availability section for more information about providers.

## Materials and equipment

• Recipes
  ○ ^az^MultiTASQ (2 mM)
    Dissolve 1.842 mg of ^az^MultiTASQ (w/ TFA) in 400 μL of ddH_2_O (*q.s.* 400 μL).
    ***Note:*** Count *ca.* 4 molecules of TFA per each TASQ molecule. See Tables 1 and 2 for information about the molar weight, exact mass (m) and the m/z ratio predicted for the ^az^MultiTASQ. After dissolution, the ^az^MultiTASQ HPLC-MS peak is at 7.8–9.6% of acetonitrile (acetonitrile increasing gradient; flow rate = 0.500 mL/min). Store at −20°C.
    **CRITICAL:** TFA is an aquatic hazard 3, corrosive 1a (skin), irritating 1 (eye) and toxic 4 (inhalation) compound (GHS05: corrosive; GHS07: harmful); wear protective lab coat, nitrile gloves and safety glasses, and work under fume hood.
  ○ BioCyTASQ (1 mM)
    Dissolve 1.43 mg of BioCyTASQ (w/ TFA) in 600 μL of ddH_2_O (*q.s.* 600 μL).
    ***Note:*** Count *ca.* 4 molecules of TFA per each TASQ molecule. See Tables 1 and 2 for information about the molar weight, exact mass (m) and the m/z ratio predicted for the BioCyTASQ. Store at 4°C.
    **CRITICAL:** TFA is an aquatic hazard 3, corrosive 1a (skin), irritating 1 (eye) and toxic 4 (inhalation) compound (GHS05: corrosive; GHS07: harmful); wear protective lab coat, nitrile gloves and safety glasses, and work under fume hood.
  ○ BioTASQ (1 mM)
    Dissolve 1.47 mg of BioTASQ (w/ TFA) in 600 μL of ddH_2_O (*q.s.* 600 μL).
    ***Note:*** Count *ca.* 4 molecules of TFA per each TASQ molecule. See Tables 1 and 2 for information about the molar weight, exact mass (m) and the m/z ratio predicted for the BioTASQ. Store at 4°C.
    **CRITICAL:** TFA is an aquatic hazard 3, corrosive 1a (skin), irritating 1 (eye) and toxic 4 (inhalation) compound (GHS05: corrosive; GHS07: harmful); wear protective lab coat, nitrile gloves and safety glasses, and work under fume hood.
  ○ Biotin-PEG(4)-DBCO (10 mM)
    Dissolve 1.5 mg of biotin-PEG(4)-DBCO in 200 μL of ddH_2_O (*q.s.* 200 μL).
    ***Note:*** See Tables 1 and 2 for information about the molar weight, exact mass (m) and the m/z ratio predicted for the biotin-PEG(4)-DBCO. After dissolution, the biotin-PEG(4)-DBCO HPLC-MS peak is at 80.2–81.3% of acetonitrile (acetonitrile increasing gradient; flow rate = 0.500 mL/min). Store at −20°C.
  ○ Biotin-clicked ^az^MultiTASQ (1 mM)
    For the preparation of biotin-clicked ^az^MultiTASQ, a Strain-Promoted Azide-Alkyne Cycloaddition (SPAAC) has to be conducted between the ^az^MultiTASQ (2 mM) and the Biotin-PEG(4)-DBCO (10 mM) (see Figure 2). To this end, a mixture of 100 μL of ^az^MultiTASQ (2 mM) (w/o TFA) with 22 μL (1.1 mol. equiv.) of biotin-PEG(4)-DBCO (10 mM) and 78 μL of ddH_2_O (*q.s.* 200 μL) is stirred at 37°C for 1 h under agitation.
    ***Note:*** See Tables 1 and 2 for information about the molar weight, exact mass (m) and the m/z ratio predicted for the biotin-clicked ^az^MultiTASQ. For checking the efficiency of the reaction: the biotin-clicked ^az^MultiTASQ HPLC-MS peak is at 55.9–57.8% of acetonitrile (acetonitrile increasing gradient; flow rate = 0.500 mL/min). Store at 4°C.
  ○ Biotin (10 mM)
    Dissolve 1.22 mg of Biotin in *ca.* 500 μL of ddH_2_O (*q.s.* 500 μL).
    ***Note:*** Store at 4°C.
  ○ Biotin (1 mM)
    Mix 60 μL of Biotin (10 mM) with 540 μL of ddH_2_O (*q.s.* 600 μL).
    ***Note:*** Store at 4°C.
  ○ BRACO-19 (2 mM)
    Dissolve 1.19 mg of BRACO-19 in *ca.* 1 mL of DMSO (*q.s.* 1 mL).
    ***Note:*** Prepare under a safety cabinet for sterility. Store at 4°C for short-term and −80°C for long-term.
    **CRITICAL:** BRACO-19 is a toxic 3 (oral) compound (GHS06: toxic); wear protective lab coat and nitrile gloves.
  ○ Denaturing agarose gel (0.026% (w/v) bleach, 1.5% (w/v) agarose**)** (25 wells 105 × 83 mm)

**Table:** 

Reagent	Final concentration	Amount
Agarose	1.5% (w/v)	1.2 g
TBE buffer (1X)	0.99X	79.2 mL
Bleach (2.6% w/v)	0.026% (w/v), 1% (v/v)	0.8 mL
Total	5X	80 mL (*ca.*)

    ***Note:*** Dissolve the agarose in TBE buffer (1X) and boil this mixture at 90°C for 10 min under agitation (or use a microwave). Then, add bleach into the mixture and boil again for 5 min. Cool down the mixture and cast it (do not forget combs!). Let the gel polymerize for 20 min at 25°C. Carefully pull the combs out and immerse the gel in the running buffer (i.e., TBE buffer (1X)). Store immersed at 4°C for up to 4 days.
    ***Note:*** You can increase the bleach concentration up to 1% (w/v) for harder denaturing condition.
    ***Note:*** If the gel is prepared in an Erlenmeyer flask, a high temperature and a slow agitation during the boiling of the gel mixture avoid bubble formation.
    **CRITICAL:** Bleach is an aquatic hazard 1 and irritating 2 (eye, skin) compound (GHS07: harmful; GHS09: environmental hazard); wear protective lab coat, nitrile gloves and safety glasses. Orthoboric acid is a toxic 1 (fœtus, germ cells) compound (GHS08: health hazard; reprotoxic R1b); wear protective lab coat and nitrile gloves. EDTA is an irritating 2 (eye), toxic 2 (respiratory system) and toxic 4 (inhalation) compound (GHS07: harmful; GHS08: health hazard); wear protective lab coat, nitrile gloves and safety glasses, and work under fume hood.
  ○ DEPC-H_2_O (0.1% v/v)
    Mix 0.5 mL of DEPC with 499.5 mL of UltraPure DNase/RNase-free distilled water (*q.s.* 500 mL).
    ***Note:*** Filter through a 0.22 μm bottle filter (Stericup) under a safety cabinet. Store at room temperature (25°C) for up to 6 months.
    **CRITICAL:** DEPC is a toxic 4 (oral) compound (GHS07: harmful); wear protective lab coat and nitrile gloves.
  ○ DEPC-PBS (0.09% v/v)
    Mix 4.5 mL of 10X PBS with 40.5 mL of DEPC-H_2_O (*q.s.* 45 mL).
    ***Note:*** Prepare this solution freshly and then store on ice.
    **CRITICAL:** DEPC is a toxic 4 (oral) compound (GHS07: harmful); wear protective lab coat and nitrile gloves.
  ○ DNase I (0.91 U/μL) (from the RNA Clean & Concentrator-5 kit)
    Reconstitute the lyophilized DNase I (250 U) with 275 μL of ddH_2_O (*q.s.* 275 μL), mix by inversion and aliquote.
    ***Note:*** Store at −20°C.
  ○ EDTA buffer (0.5 M, pH 8.0)
    Dissolve 4.38 g of EDTA in *ca.* 30 mL of ddH_2_O (*q.s.* 30 mL).
    ***Note:*** Adjust the pH to 8.0 with NaOH (1 M). Filter through a 0.2 μm syringe filter under a safety cabinet. Store at 4°C for up to 6 months.
    **CRITICAL:** EDTA is an irritating 2 (eye), toxic 2 (respiratory system) and toxic 4 (inhalation) compound (GHS07: harmful; GHS08: health hazard); wear protective lab coat, nitrile gloves and safety glasses, and work under fume hood.
  ○ EGTA buffer (0.5 M, pH 8.0)
    Dissolve 5.71 g of EGTA in *ca.* 30 mL of ddH_2_O (*q.s.* 30 mL).
    ***Note:*** Adjust the pH to 8.0 with NaOH (1 M). Filter through a 0.2 μm syringe filter under a safety cabinet. Store at 4°C for up to 6 months.
  ○ Ethanol (70% v/v)
    Mix 7 mL of Ethanol absolute with 3 mL of ddH_2_O (*q.s.* 10 mL).
    ***Note:*** Prepare this solution freshly and then store at 25°C.
    **CRITICAL:** Ethanol absolute is a flammable 2 and irritating 2 (eye) compound (GHS02: flammable; GHS07: harmful); wear protective lab coat, nitrile gloves and safety glasses, and keep away from heat source.
  ○ Fixing buffer (5X)

**Table:** 

Reagent	Final concentration	Amount
HEPES KOH buffer (1 M, pH 7.5)	250 mM	25 mL
NaCl (1 M)	500 mM	50.0 mL
EDTA (0.5 M, pH 8.0)	5 mM	1.0 mL
EGTA (0.5 M, pH 8.0)	2.5 mM	0.5 mL
DEPC-H_2_O (0.1% v/v)	N/A	23.5 mL
Total	5X	100 mL

    ***Note:*** Filter through a 0.2 μm syringe filter under a safety cabinet. Store at 4°C for up to 6 months.
    **CRITICAL:** KOH is a corrosive 1 (metals, skin), irritating 1 (eye) and toxic 4 (oral) compound (GHS05: corrosive; GHS07: harmful); wear protective lab coat, nitrile gloves and safety glasses. EDTA is an irritating 2 (eye), toxic 2 (respiratory system) and toxic 4 (inhalation) compound (GHS07: harmful; GHS08: health hazard); wear protective lab coat, nitrile gloves and safety glasses, and work under fume hood. DEPC is a toxic 4 (oral) compound (GHS07: harmful); wear protective lab coat and nitrile gloves.
  ○ Formaldehyde (1% w/v)/Fixing buffer (1X)

**Table:** 

Reagent	Final concentration	Amount
Formaldehyde (16% w/v)	1% (w/v)	1.9 mL
Fixing buffer (5X)	1X	6.0 mL
DEPC-H_2_O (0.1% v/v)	N/A	22.1 mL
Total	N/A	30 mL

    ***Note:*** Prepare this solution freshly and then store on ice in protecting it from light.
    **CRITICAL:** Formaldehyde is a flammable 3, corrosive 1b, toxic 1 (central nervous and respiratory systems) and toxic 3 (dermal, inhalation, oral) compound (GHS07: harmful; GHS08: health hazard); wear protective lab coat, nitrile gloves and safety glasses, and work under fume hood. KOH is a corrosive 1 (metals, skin), irritating 1 (eye) and toxic 4 (oral) compound (GHS05: corrosive; GHS07: harmful); wear protective lab coat, nitrile gloves and safety glasses. EDTA is an irritating 2 (eye), toxic 2 (respiratory system) and toxic 4 (inhalation) compound (GHS07: harmful; GHS08: health hazard); wear protective lab coat, nitrile gloves and safety glasses, and work under fume hood. DEPC is a toxic 4 (oral) compound (GHS07: harmful); wear protective lab coat and nitrile gloves.
  ○ G4RP buffer (pH 7.4)

**Table:** 

Reagent	Final concentration	Amount
Trizma base	25 mM	181.71 mg
KCl (1 M)	150 mM	9.0 mL
EDTA (0.5 M, pH 8.0)	5 mM	0.6 mL
DTT (10 mM)	0.5 mM	3.0 mL
Tergitol (70% w/v)	0.35% (w/v), 0.5% (v/v)	0.3 mL
DEPC-H_2_O (0.1% v/v)	N/A	47.1 mL (*ca.)*
Total	N/A	60 mL

    ***Note:*** Adjust pH to 7.4 with HCl (1 M). Filter through a 0.2 μm syringe filter under a safety cabinet. Store at 4°C for up to 6 months.
    **CRITICAL:** EDTA is an irritating 2 (eye), toxic 2 (respiratory system) and toxic 4 (inhalation) compound (GHS07: harmful; GHS08: health hazard); wear protective lab coat, nitrile gloves and safety glasses, and work under fume hood. DTT is a corrosive 1 (eye), irritating 2 (skin) and toxic 4 (oral) compound (GHS05: corrosive; GHS07: harmful); wear protective lab coat, nitrile gloves and safety glasses. Tergitol is an aquatic hazard 2, corrosive and toxic 4 (inhalation, oral) compound (GHS05: corrosive; GHS07: harmful; GHS09: environmental hazard); wear protective lab coat, nitrile gloves and safety glasses, and work under fume hood. DEPC is a toxic 4 (oral) compound (GHS07: harmful); wear protective lab coat and nitrile gloves.
  ○ G4RP lysis buffer

**Table:** 

Reagent	Final concentration	Amount
RNase OUT	250 μM, 0.1 U/μL	3.75 μL
SDS (20% w/v)	0.1% (w/v), 0.5% (v/v)	7.50 μL
G4RP buffer (pH 7.4)	N/A	1,488.75 μL (*ca.)*
Total	N/A	60 mL

    ***Note:*** Prepare this solution freshly. Mix by leading the tube with hands. Store at 4°C (not on ice, to avoid SDS precipitation).
    **CRITICAL:** EDTA is an irritating 2 (eye), toxic 2 (respiratory system) and toxic 4 (inhalation) compound (GHS07: harmful; GHS08: health hazard); wear protective lab coat, nitrile gloves and safety glasses, and work under fume hood. DTT is a corrosive 1 (eye), irritating 2 (skin) and toxic 4 (oral) compound (GHS05: corrosive; GHS07: harmful); wear protective lab coat, nitrile gloves and safety glasses. SDS is an aquatic hazard 3, corrosive 1 (eye), flammable 2, irritating 2 (skin), toxic 3 (respiratory system) and toxic 4 (inhalation, oral) compound (GHS02: flammable; GHS05: corrosive; GHS07: harmful); wear protective lab coat, nitrile gloves and safety glasses, work under fume hood and keep away from heat source. Tergitol is an aquatic hazard 2, corrosive and toxic 4 (inhalation, oral) compound (GHS05: corrosive; GHS07: harmful; GHS09: environmental hazard); wear protective lab coat, nitrile gloves and safety glasses, and work under fume hood. DEPC is a toxic 4 (oral) compound (GHS07: harmful); wear protective lab coat and nitrile gloves.
  ○ G4RP wash buffer
    Mix 9 μL of RNase OUT with 2391 μL of G4RP buffer (pH 7.4) (*q.s.* 2.4 mL).
    ***Note:*** Prepare this solution freshly and then store at 4°C
    **CRITICAL:** EDTA is an irritating 2 (eye), toxic 2 (respiratory system) and toxic 4 (inhalation) compound (GHS07: harmful; GHS08: health hazard); wear protective lab coat, nitrile gloves and safety glasses, and work under fume hood. DTT is a corrosive 1 (eye), irritating 2 (skin) and toxic 4 (oral) compound (GHS05: corrosive; GHS07: harmful); wear protective lab coat, nitrile gloves and safety glasses. Tergitol is an aquatic hazard 2, corrosive and toxic 4 (inhalation, oral) compound (GHS05: corrosive; GHS07: harmful; GHS09: environmental hazard); wear protective lab coat, nitrile gloves and safety glasses, and work under fume hood. DEPC is a toxic 4 (oral) compound (GHS07: harmful); wear protective lab coat and nitrile gloves.
  ○ Glycine (1 M)
    Dissolve 3.38 g of glycine in *ca.* 45 mL of DEPC-H_2_O (0.1% v/v) (*q.s.* 45 mL).
    ***Note:*** Filter through a 0.2 μm syringe filter under a safety cabinet. Store at 4°C for up to 6 months.
  ○ HCl (1 M)
    Mix 8.35 mL of HCl (37% m/m) with 91.65 mL of ddH_2_O (*q.s.* 100 mL).
    ***Note:*** Add firstly the HCl, then the ddH_2_O. Store at room temperature (25°C).
    **CRITICAL:** HCl is a corrosive 1 (metals, skin), irritating 1b (eye, skin) and toxic 3 (respiratory system) compound (GHS05: corrosive; GHS07: harmful); wear protective lab coat, nitrile gloves and safety glasses, and work under fume hood.
  ○ HEPES KOH buffer (1 M, pH 7.5)
    Dissolve 47.66 g of HEPES in *ca.* 200 mL of ddH_2_O (*q.s.* 200 mL).
    ***Note:*** Adjust pH to 7.5 with KOH (1 M) (*ca.* 20–30 mL). Filter through a 0.2 μm syringe filter under a safety cabinet. Store at 4°C for up to 3 months.
    **CRITICAL:** KOH is a corrosive 1 (metals, skin), irritating 1 (eye) and toxic 4 (oral) compound (GHS05: corrosive; GHS07: harmful); wear protective lab coat, nitrile gloves and safety glasses.
  ○ KCl (1 M)
    Dissolve 7.46 g of KCl in *ca.* 100 mL of ddH_2_O (*q.s.* 100 mL).
    ***Note:*** Store at 4°C for up to 1 year.
  ○ KOH (1 M)
    Dissolve 11.22 g of KOH in *ca.* 200 mL of ddH_2_O (*q.s.* 200 mL).
    ***Note:*** Store at 4°C for up to 1 year.
    **CRITICAL:** KOH is a corrosive 1 (metals, skin), irritating 1 (eye) and toxic 4 (oral) compound (GHS05: corrosive; GHS07: harmful); wear protective lab coat, nitrile gloves and safety glasses.
  ○ NaCl (1 M)
    Dissolve 5.84 g of NaCl in *ca.* 100 mL of ddH_2_O (*q.s.* 100 mL).
    ***Note:*** Store at 4°C for up to 1 year.
  ○ NaOH (1 M)
    Dissolve 4.0 g of NaOH in *ca.* 100 mL of ddH_2_O (*q.s.* 100 mL).
    ***Note:*** Store at room temperature (25°C).
    **CRITICAL:** NaOH is a corrosive 1 (metals, skin) and irritating 1a (eye, skin) compound (GHS05: corrosive; GHS07: harmful); wear protective lab coat, nitrile gloves and safety glasses.
  ○ PhpC (20 mM)
    Dissolve 3.56 mg of PhpC in *ca.* 500 μL of ddH_2_O (*q.s.* 500 μL).
    ***Note:*** Prepare under a safety cabinet for sterility. Store at 4°C for short-term and −20°C for long-term.
  ○ qPCR primers couples (10 μM)
    Dissolve the qPCR primers at high concentration (e.g., 500 μM) and check their concentration. Dilute them at 10 μM with UltraPure DNase/RNase-free distilled water.
    ***Note:*** Store at 4°C for up to 1 year.
    **CRITICAL:** The use of an UltraPure DNase/RNase-free distilled water is critical for the RT-qPCR to avoid contamination and/or degradation of purified samples.
  ○ RNA Wash Buffer (from the RNA Clean & Concentrator-5 kit)
    Mix 24 mL of RNA Wash Buffer concentrate with 96 mL of Ethanol absolute (*q.s.* 120 mL).
    ***Note:*** Store at room temperature (25°C) for up to 1 year.
  ○ SDS (20% w/v)
    Dissolve 10.0 g of SDS in *ca.* 50 mL of ddH_2_O (*q.s.* 50 mL).
    ***Note:*** Store at 25°C for up to 6 months. If it precipitates, warm it at 37°C and resuspend slowly.
    **CRITICAL:** SDS is an aquatic hazard 3, corrosive 1 (eye), flammable 2, irritating 2 (skin), toxic 3 (respiratory system) and toxic 4 (inhalation, oral) compound (GHS02: flammable; GHS05: corrosive; GHS07: harmful); wear protective lab coat, nitrile gloves and safety glasses, work under fume hood and keep away from heat source.
  ○ Supplemented DMEM culture medium

**Table:** 

Reagent	Final concentration	Amount
DMEM	N/A	500 mL
FBS	10% (v/v),	56 mL
Pen-Strep	1% (v/v), 106.8 U/mL (Pen), 106.8 μg/mL (Strep)	6 mL
Total	N/A	562 mL

    ***Note:*** Prepare under a safety cabinet for sterility. Store at 4°C.
    **CRITICAL:** Pen-Strep is a toxic 2 (germ cells) compound (GHS08: health hazard; reprotoxic R2); wear protective lab coat and nitrile gloves, and work under safety cabinet
  ○ TBE buffer (10X, pH 8.3)

**Table:** 

Reagent	Final concentration	Amount
Trizma base	891.5 mM	108.0 g
Orthoboric acid	889.5 mM	55.0 g
EDTA	31.8 mM	9.3 g
ddH_2_O	N/A	1 L (*ca.*)
Total	10X	562 mL

    ***Note:*** Adjust pH to 8.3 with HCl (1 M). Store at room temperature (25°C) for up to 1 year.
    **CRITICAL:** Orthoboric acid is a toxic 1 (fœtus, germ cells) compound (GHS08: health hazard; reprotoxic R1b); wear protective lab coat and nitrile gloves. EDTA is an irritating 2 (eye), toxic 2 (respiratory system) and toxic 4 (inhalation) compound (GHS07: harmful; GHS08: health hazard); wear protective lab coat, nitrile gloves and safety glasses, and work under fume hood.
  ○ TBE buffer (1X)
    Mix 100 mL of TBE buffer (10X, pH 8.3) with 900 mL of ddH_2_O (*q.s.* 1 L).
    ***Note:*** Store at 4°C for up to 3 months.
    **CRITICAL:** Orthoboric acid is a toxic 1 (fœtus, germ cells) compound (GHS08: health hazard; reprotoxic R1b); wear protective lab coat and nitrile gloves. EDTA is an irritating 2 (eye), toxic 2 (respiratory system) and toxic 4 (inhalation) compound (GHS07: harmful; GHS08: health hazard); wear protective lab coat, nitrile gloves and safety glasses, and work under fume hood.

• Equipment setup
  ○ Reverse Transcription (RT) and real-time PCR (qPCR) programs
    For the steps of RT and qPCR at the end of the G4RP.v2 protocol (Figure 3A), two procedures were created in a real-time PCR system and thermocycler (Agilent) (Figures 3B and 3C; Table 3).
    ***Note:*** 60 cycles for qPCR amplification were chosen during the first experiments in order to set up the protocol and assess the influence of the optimizations made on the RT-qPCR results. In order to obtain the Ct value of some controls (e.g., without cDNA sample or control precipitation with biotin) the number of cycles has to be slightly over 30. The number of cycles can be reasonably reduced from 60 to 35.

• Alternative materials and equipment
  ○ Cell lysis
    The cell lysis in the G4RP.v2 protocol is performed using a needle-equipped syringe for cell membrane and nuclear envelope destruction. Alternatively, a cell scraper or a douncer can be considered: the cell scraper allows for the complete destruction of membranes and is an easy and cheap method; the douncer (or dounce homogenizer) is also an efficient way to lyse cells but has to be combined with a strong lysis buffer (with ionic detergent) to recover the nuclear components, otherwise only the cytoplasmic RNAs will be taken. In the case of experiments with several conditions (e.g., different types of treatment), the douncer method is at risk regarding cross-contaminations between samples if the glass material (i.e., the cylindrical mortar and the pestle) is not washed with sufficiently stringent solutions. These two alternative lysis methods can also be combined with sonication.

## Step-by-step method details

### Cell seeding and preparation for G4-interacting agent treatments

**Timing: 1 night + 2 days**

The second phase of the G4RP.v2 protocol, the cell culture and treatment phase, intervenes after the previously described preparation phase. Here, the procedure to prepare MCF7 cells in culture flask and their incubation (or not) with G4-interacting agents (BRACO-19 or PhpC) is detailed.

1. On day 1 (afternoon), seed 7 × 10^6^ MCF7 cells (in exponential phase growth) in supplemented DMEM culture medium (*q.s.* 8 mL) per culture flask (175 cm^2^).
  a. Allow MCF7 cells to recover overnight under cell culture condition (37°C, 5% CO_2_).

***Note:*** Perform a preliminary growth test to check whether the seeded density is enough to obtain around 20 × 10^6^ cells on day 4 (morning) for the third phase of the protocol. Also, optimization experiments (see the resource availability section) showed that working with 10 × 10^6^ cells could be sufficient (see the expected outcomes section).

***Note:*** 8 mL of medium is sufficient for cell growth and allow for saving G4-interacting agents for subsequent treatment.

***Note:*** One culture flask can be prepared for non-treated cells (control) and two others for G4-interacting agents-treated cells (i.e., with BRACO-19 or PhpC).

***Optional:*** To avoid losing time in counting cells in all culture flasks during the next step (cell lysis), prepare another culture flask in the same conditions, which will be used for cell counting only.

**CRITICAL:** Depending on cell lines and RNA targets, the number of needed cells may vary.

2. On day 2 (morning), remove the medium and keep them separately.
  a. Add the appropriate G4-interacting agent (or not) into the respective kept medium (see problem 1, troubleshooting section):
    i. Add 33.7 μL of DMSO (*ca.* 100%) in 8 mL of medium for the non-treated cell condition.
    ii. Add 33.7 μL of BRACO-19 (2 mM, in DMSO) in 8 mL of medium for the BRACO-19-treated cell condition (final concentration of BRACO-19 = 8.42 μM or 5 μg/mL).
    iii. Add 36 μL of PhpC (20 mM, in ddH_2_O) and 33.7 μL of DMSO (*ca.* 100%) in 8 mL of medium for the PhpC-treated cell condition (final concentration of PhpC = 90 μM).
  b. Homogenize the medium.
  c. Put the treatment-complemented medium back to the appropriate culture flask.
  d. Incubate cells for 48 h under normal cell culture condition (37°C, 5% CO_2_).

***Note:*** If a G4-interacting agent is dissolved in DMSO, make sure to keep the same percentage (% v/v) of DMSO for all condition (here, 0.4% v/v).

**CRITICAL:** If a G4-interacting agent treatment is used, a dose-finding experiment should be performed in order to select a suitable dose, that is, a non-toxic or low-toxicity dose (< IC_20_) as a working concentration.

### Cell crosslinking to preserve biological interactions

**Timing: 1–2 h**

The third phase of the protocol, which is the G4RP.v2 protocol *per se*, intervenes after the previously described cell growing and treatment phase. Here, the procedure to count, cross-link and wash MCF7 cells is described. This step will lead to fixated cell pellets suitable for lysis. Perform this whole step in an ice-filled box (unless otherwise indicated).

3. On day 4 (morning), prepare fresh buffers and solutions: DEPC-PBS (0.09% v/v), ethanol (70% v/v), Formaldehyde (1% w/v)/Fixing buffer (1X), G4RP lysis buffer and G4RP wash buffer.
  a. Store them at 4°C, but the ethanol at room temperature (25°C) for day 5.
4. Detach cells with 4 mL of trypsin.
  a. Add an equal volume of supplemented DMEM culture medium.
  b. Put this cell suspension in 15 mL tubes.
    i. Homogenize it.
    ii. Take 10 μL for counting cells.
    iii. Put the tubes under normal cell culture condition (37°C, 5% CO_2_) during the counting step.
  c. Count cells rapidly with a cell counting chamber to make sure that the subsequent experiments will be performed with *ca.* 20 × 10^6^ cells (see problem 2, troubleshooting section).

***Note:*** See the Cell preparation sub-section in the before you begin section to have details on trypsin treatment.

***Note:*** Cells can be directly crosslinked without any counting step because the 5% input control will allow for the normalization of data for every condition. However, a cell number reference is useful to be sure there was no problem during the cell growing (see the CRITICAL and Optional callouts above).

**CRITICAL:** If you want to count every flask, do it in a very fast manner and keep the 15 mL tubes under cell culture condition (37°C, 5% CO_2_).

***Optional:*** If you prepared another culture flask solely for counting cells, count the cells inside of this flask only and consider the cell number as equivalent for the others flasks.

5. Crosslink cells:
  a. Centrifuge at 1 500 rpm (210 G) for 5 min at 4°C.
    i. Remove slowly the supernatant.
  b. Resuspend the cell pellet in 10 mL of cold DEPC-PBS.
  c. Centrifuge at 1 500 rpm (210 G) for 5 min at 4°C.
    i. Remove the PBS.
  d. Add 10 mL of cold Formaldehyde (1% w/v)/Fixing buffer (1X).
    i. Vortex well.
    ii. Incubate on a benchtop laboratory rocker for 5 min at room temperature (25°C).

**CRITICAL:** The duration of the formaldehyde crosslinking step is important.

**CRITICAL:** After crosslinking, keep the sample at 4°C as much as possible (even during centrifugation).

***Optional:*** The incubation in Formaldehyde (1% w/v)/Fixing buffer (1X) can be manually done (instead of using a rocker).

6. Quench crosslink cells:
  a. Add 1.43 mL of glycine (1 M) to a final concentration of 125 mM.
    i. Vortex well.
    ii. Incubate on a benchtop laboratory rocker for 5 min at room temperature (25°C).
7. Wash cells:
  a. Centrifuge at 1 500 rpm (210 G) for 5 min at 4°C.
    i. Remove the fixing solution.
  b. Resuspend the cell pellet in 0.8 mL of DEPC-PBS.
  c. Transfer into 1.5 mL microtubes.
  d. Centrifuge at 1 500 rpm (210 G) for 3 min at 4°C.
    i. Remove the supernatant.
  e. Resuspend the cell pellet in 1 mL of DEPC-PBS.
  f. Centrifuge at 1 500 rpm (210 G) for 3 min at 4°C.
    i. Remove the supernatant.

**CRITICAL:** The 3-min centrifugation steps allow for accumulating the cell pellets along the corner of the tube, which facilitates the aspiration of the supernatant.

### Cell lysis for recovering total RNA

**Timing: 2–3 h**

This step describes the procedure to lyse cells with a needle-equipped syringe. This step generates cell lysates which will be subsequently used for the key G4 precipitation step. Perform this whole step in an ice-filled box (unless otherwise indicated).

8. Resuspend the cell pellet in 400 μL of G4RP lysis buffer.
9. Make 100 quite fast pipetting (up-and-down) with a 0.4 mm needle-equipped syringe (see problem 3, troubleshooting section).

***Note:*** Quick spin the tube on a regular basis (i.e., every 20 up-and-downs) to homogenize and optimize the lysis.

**CRITICAL:** Depending on the number of cells, the aspiration can be very slow, do not hesitate to force.

**CRITICAL:** If buffer and/or bubble pass through the gasket to the piston, disassemble this part of the syringe and aspirate the buffer with a pipette in order to not lose any cells/lysate.

10. Centrifuge at 13 200 rpm (16 550 G) for 10 min at 4°C.
11. Recover the supernatant of this raw lysate to a new 1.5 mL microtube.

**CRITICAL:** You can already check the concentration of samples to assess the efficiency of cell lysis (see the UV absorbance measurement step below).

**CRITICAL:** Make a denaturing agarose gel electrophoresis (see the Gel electrophoresis step below) and search for the DNA high band to be sure that you have efficiently lysed cells and *nuclei* (which is critical if you plan to study nuclear RNAs).

**Pause point:** It is, in theory, possible to freeze the raw lysates (supernatants) at this point for later processing (but it has never been attempted).

### Precipitation of G4s from cell lysates using TASQs

**Timing: 5–5.5 h**

This step describes the procedure to extract G4s (DNA and RNA) from cell lysates in using biotinylated TASQs: the procedure to precipitate G4s, wash and recover these samples, and to reverse the crosslinking is presented. This step will lead to samples of G4s comprising the G4-RNA of interest. Perform this whole step in an ice-filled box (unless otherwise indicated).

12. Homogenize the raw lysate supernatant (around 380 μL).
  a. Aliquot into 0.2 mL PCR tubes as following:
    i. 60 μL for the precipitation control with biotin.
    ii. 60 μL for the G4-precipitation with a TASQ.
    iii. 3 μL for the 5% input control (i.e., 5% of the starting material of G4-precipitation).
    iv. 8 μL for monitoring techniques (i.e., RNA quantification by UV absorbance measurement and RNA quality check by gel electrophoresis).

***Note:*** This volume (60 μL) was selected because this method was performed with 3 different TASQs concomitantly (BioTASQ, BioCyTASQ and biotin-clicked ^az^MultiTASQ); it can be adjusted depending on the implemented experimental conditions and the abundance of the RNA of interest.

***Optional:*** For monitoring the lysis step, reverse crosslink (*vide infra*) directly the raw lysate supernatant sample (8 μL) to process it then by the two monitoring techniques.

13. Store temporarily the 5% input tube at 4°C.
14. Homogenize slowly the Streptavidin MagneSphere Paramagnetic Particles.

**CRITICAL:** Use wide-mouth tips to gently resuspend the streptavidin-coated magnetic particles.

15. Put in a 1.5-mL microtube the volume of magnetic particles necessary for the experiment.

***Note:*** The amount of particles can be optimized depending on the abundance of RNA of interest.

16. Wash the Streptavidin MagneSphere Paramagnetic Particles with DEPC-PBS thrice:
  a. Magnet the particles.
    i. Remove the supernatant.
  b. Resuspend the particles in 500 μL of DEPC-PBS.
    i. Magnet the particles.
    ii. Remove the supernatant.
  c. Resuspend the particles in 500 μL of DEPC-PBS.
    i. Magnet the particles.
    ii. Remove the supernatant.
  d. Resuspend the particles in 500 μL of DEPC-PBS.
    i. Magnet the particles.
    ii. Remove the supernatant.

**CRITICAL:** For washing the particles, use a magnetic rack to immobilize them at the bottom of the tube and aspirate the buffer gently; then, use wide-mouth tips to add buffer and homogenize the solution of particles.

17. Resuspend the MagneSphere particles in 100 μL of DEPC-PBS to obtain a 6 μg/μL working solution.
18. Add 15 μL of the MagneSphere particles working solution (= 90 μg) in the previous PCR tubes containing raw lysate supernatants.
19. Add 6.5 μL of biotin (1 M) or TASQ (1 M) in the appropriate PCR tubes and MagneSphere particles to a final concentration of 80 μM.

***Note:*** A volume of 81.5 μL for precipitation is fine but the final volume can be increased to *ca.* 200 μL if needed.

***Note:*** The concentration of TASQ (and of the biotin control) can be optimized depending on the abundance of RNA of interest.

20. Incubate the samples (i.e., the mixture with raw lysate supernatant, MagneSphere particles and biotin or TASQ) for 2 h at 4°C on a benchtop rotator.

***Note:*** The rotator parameters used are: orbital rotation of 35 rpm for 20 sec, then quick reciprocal vibration at 5° for 10 sec (with a 90° change after 5 sec).

**Pause point:** It is, in theory, possible to perform the incubation overnight (but it has never been attempted).

21. Recover the G4-enriched samples:
  a. Magnet the particles of the PCR tubes using magnetic rack for at least 2 min.
  b. Remove gently the supernatant (which contains unbound material).

***Optional:*** For experimental optimization, this supernatant can be kept and compared with the bound material fraction by gel electrophoresis.

22. Wash the Streptavidin MagneSphere Paramagnetic Particles:
  a. Add 200 μL of G4RP wash buffer.
    i. Homogenize gently the particles.
    ii. Incubate for 5 min at room temperature (25°C) on a benchtop rotator.
    iii. Magnet the particles.
    iv. Remove the supernatant.
  b. Add 200 μL of DEPC-PBS.
    i. Homogenize gently the particles.
    ii. Incubate for 5 min at room temperature (25°C) on a benchtop rotator.
    iii. Magnet the particles.
    iv. Remove the supernatant.

***Note:*** More washing steps can be added if necessary.

**CRITICAL:** For washing the particles, use a magnetic rack to immobilize them at the bottom of the tube and aspirate the buffer gently; then, use wide-mouth tips to add buffer and homogenize the solution of particles.

***Optional:*** For experimental optimization, these two supernatants can be kept and compared with the bound material fraction by gel electrophoresis.

23. Resuspend the particles in 100 μL of DEPC-PBS.
  a. Add 1 μL of RNase OUT in each PCR tube.
24. Take out the 5% input PCR tube previously stored at 4°C.
  a. Add 100 μL of DEPC-PBS in this 5% input PCR tube.
  b. Add 1 μL of RNase OUT in this 5% input PCR tube.
25. Transfer the samples in new 1.5-mL microtubes.
26. Reverse crosslink the samples:
  a. Resuspend well the particles.
  b. Incubate all microtubes for 2 h at 70°C on a benchtop heating block under stirring.

**CRITICAL:** The reverse crosslinking duration is determinant for the RNA accessibility and the purity of the sample.

### In-column RNA purification for purifying RNA G4s

**Timing: 1 night + 5–6 h**

This step describes the procedure of RNA purification from G4-enriched samples using an in-column RNA purification kit. This step will lead to purified RNA samples suitable for RT-qPCR quantification.

27. Add 1 mL of TRIzol as fast as possible in each microtube and mix gently.

**CRITICAL:** For your safety, manipulate TRIzol with caution under a standard fume hood.^43^

28. Quick-freeze samples with liquid nitrogen:
  a. Dive the samples in liquid nitrogen for 1 min.
  b. Store them overnight at −80°C.

***Note:*** Samples can be stored for long term in the TRIzol solution.

29. On day 5 (morning), thaw samples for 5–6 min at 27°C on benchtop heating block under gentle stirring.
  a. Mix gently by inverting tubes several times to help the thawing.
  b. Let the samples incubate with the TRIzol for 5 min at room temperature (once totally thawed) to dissociate any ribonucleoprotein complexes from G4s.

**CRITICAL:** Because the TRIzol/sample mixture leaks easily from the microtubes, keep cleaned gloves.

30. Add 0.2 mL of chloroform in each microtube.
  a. Make sure the caps are properly sealed.
  b. Shake them vigorously for 15 s at room temperature (25°C).
  c. Incubate them for 3 min at room temperature (25°C).

**CRITICAL:** Because the TRIzol/chloroform mixture leaks easily from the microtubes, place microtubes altogether on a rack and a paper towel between your hands and the microtubes to shake all of them simultaneously and avoid the splashing of the hazardous mixture on the working area.

31. Centrifuge at 12 000 rpm (13 680 G) for 15 min at 4°C.

***Note:*** The mixture will separate in 3 phases: the transparent top aqueous phase containing the RNAs, the middle white-colored interphase containing the DNA, and the bottom pink-colored organic phase containing the proteins. The interphase is not always visible, depending on the quantity of material used, especially after a precipitation step during which only a fraction of the raw lysate is bound.

***Optional:*** It is, in theory, possible to recover the DNA G4s at this step if sonication was performed after the lysis step (but it has never been attempted).

32. Maintain the samples on ice.
33. Transfer cautiously the transparent top aqueous phase (*ca.* 600 μL) into new RNase-free 1.5-mL microtubes (see problem 4, troubleshooting section).
34. Add 1 volume of absolute ethanol to 1 volume of aqueous phase (1:1).
  a. Mix well by manually inverting microtubes.
35. Proceed to the RNA purification with the RNA Clean & Concentrator-5 kit^44^ at room temperature (25°C) in following the manufacturer’s protocol:
  a. Place Zymo-Spin IC Columns over Collection Tubes (or any RNase-free cap-free 2-mL microtubes)
  b. Transfer the samples in these columns.
    i. Centrifuge at 12 000 rpm (13 680 G) for 30 s at room temperature (25°C).
    ii. Discard the flow-through (the phase in the bottom microtube).
  c. Add 400 μL of RNA Wash Buffer to the columns.
    i. Centrifuge at 12 000 rpm (13 680 G) for 30 s at room temperature (25°C).
    ii. Discard the flow-through.
  d. Prepare the DNase I Reaction Mix:
    i. Add 5 μL of reconstituted *ca.* 1 U/μL DNase I to 35 μL of DNA Digestion Buffer in a RNase-free tube for each sample.
    ii. Mix by gentle inversion.
    iii. Add 40 μL of the DNase I Reaction Mix directly into the column matrix.
    iv. Incubate for 15 min at room temperature (25°C).
  e. Add 400 μL of RNA Prep Buffer to the column.
    i. Centrifuge at 12 000 rpm (13 680 G) for 30 s at room temperature (25°C).
    ii. Discard the flow-through.
  f. Add 700 μL of RNA Wash Buffer to the column.
    i. Centrifuge at 12 000 rpm (13 680 G) for 30 s at room temperature (25°C).
    ii. Discard the flow-through.
  g. Add 400 μL of RNA Wash Buffer to the column.
    i. Centrifuge at 12 000 rpm (13 680 G) for 30 s at room temperature (25°C).
    ii. Discard the flow-through.
    iii. Ensure the complete removal of the RNA Wash Buffer.
  h. Transfer carefully the columns in new RNase-free 1.5-mL microtubes.
  i. Add 15 μL of UltraPure DNase/RNase-free distilled water directly in the column matrix.
    i. Incubate 2 min.
    ii. Centrifuge at 12 000 rpm (13 680 G) for 30 s at room temperature (25°C).
    iii. Keep the flow-through in the bottom microtube (= 15 μL).
  j. Add again 15 μL of UltraPure DNase/RNase-free distilled water directly in the column matrix.
    i. Incubate 2 min.
    ii. Centrifuge at 12 000 rpm (13 680 G) for 30 s at room temperature (25°C).
    iii. Keep the flow-through in the bottom microtube (= 30 μL).

***Note:*** The maximum volume of the Zymo-Spin IC Column is 700 μL, so repeat the step 35-b several times if necessary, in refilling the same column.

**CRITICAL:** The maximum binding capacity of the Zymo-Spin IC Columns is supposed to be 10 μg RNA but 2.6 μg RNA were recovered in our hands, which is enough for our study but can be improved for less abundant RNA targets. To get more RNA, the same top aqueous phase can be separated in two columns (and then the two purified RNA tubes be gathered) or the Zymo-Spin IIC Columns which have a binding capacity of 50 μg RNA can be used.

36. Incubate samples for 15 min at 55°C on a benchtop heating block for RNA resolubilization.
  a. Keep purified RNA samples on ice.
  b. Check the RNA concentration and purity by UV absorbance measurement (see problems 5 and 6, troubleshooting section).

**Pause point:** Purified RNA samples can be stored up to 1 year at −80°C in RNase-free metals-chelating buffers (e.g., EDTA) to avoid RNase activity (but it has never been attempted, the samples were processed directly).

**CRITICAL:** Avoid repeating freeze/thaw cycles.

### RT and qPCR quantification of the purified *NRAS* and *VEGFA* mRNAs

**Timing: 4–5 h**

This step describes the procedure to reverse transcribe the purified RNA in cDNA and then, to quantify the amount of *NRAS* and *VEGFA* cDNAs by qPCR. This step will lead to PCR data relative to the direct quantity of these two cDNAs and then, to the indirect quantity of the two corresponding *NRAS* and *VEGFA* mRNAs. Perform this whole step in an ice-filled box (unless otherwise indicated).

***Note:*** The following RT reaction performed in 20 μL can be used for 10 pg-5 μg of total RNA or 10 pg-500 ng of mRNA (according to the SuperScript III Reverse Transcriptase provider).

**CRITICAL:** Mix preferentially stock solutions and master mixes by gently flicking the tube or pipetting up-and-down to preserve RNA and product integrity. Vortex is usually not recommended but one very short (< 0.5 sec) vortexing should be fine to help homogenizing.

37. Proceed to the hybridization reaction:
  a. Make a master mix A with the following components:
    i. 1 μL of Random hexamers (50 μM).
    ii. 1 μL of dNTP mix (10 mM).
  b. Mix gently.
  c. Add the master mix A to 12.5 μL of purified RNA.
  d. Mix gently.
  e. Incubate for 5 min at 65°C.
  f. Place on ice for 5 min.

**Table:** 

Reagent	Final concentration	Amount
Random hexamers (50 μM)	3.45 μM	1 μL
dNTP mix (10 mM)	689.7 μM	1 μL
Purified RNA (variable concentration)	Variable concentration	12.5 μL
Total	N/A	14.5 μL

38. Proceed to the Reverse Transcription (RT) reaction:
  a. Make a master mix B with the following components:
    i. 4 μL of First Strand Buffer (5X).
    ii. 1 μL of DTT (0.1 M).
    iii. 0.25 μL of RNase OUT (100 mM).
    iv. 0.25 μL of SuperScript III Reverse Transcriptase (200 U/μL).^45^
  b. Mix gently.
  c. Do a very brief centrifugation if necessary.
  d. Incubate for 5 min at room temperature (25°C).
  e. Incubate for 45 min at 55°C.
  f. Incubate for 15 min at 70°C to inactive the RT reaction.
  g. Cool down to room temperature (25°C).

**Table:** 

Reagent	Final concentration	Amount
Random hexamers (50 μM)	2.5 μM	1 μL
dNTP mix (10 mM)	500 μM	1 μL
Purified RNA (variable concentration)	Variable concentration	12.5 μL
First Strand Buffer (5X)	1X	4 μL
DTT (0.1 M)	5 mM	1 μL
RNase OUT (100 mM)	1.25 mM	0.25 μL
SuperScript III RT (200 U/μL)	2.5 U/μL	0.25 μL
Total	N/A	20 μL

***Note:*** Use the RT program written on the software of the used thermocycler to make this RT step easier and more reproducible (see the programs preparation sub-section in the before you begin section, Figures 3A and 3B; Table 3).

**Pause point:** The cDNA samples can be stored up to 1 year at −80°C (but it has never been attempted, the samples were processed directly). Avoid repeated freeze/thaw cycles.

39. Proceed to the qPCR reaction with the appropriate primer set:
  a. Make a master mix C with the following components for each primers couple:
    i. 0.5 μL of forward primer (10 μM).
    ii. 0.5 μL of reverse primer (10 μM).
    iii. 5 μL of i*Taq* Universal SYBR Green Supermix (2X).^46^
    iv. 3 μL of UltraPure DNase/RNase-free Distilled water.
  b. Mix gently.
  c. Add 9 μL of appropriate master mix C in a 96-well PCR plate.
  d. Add 1 μL of the appropriate sample to the appropriate plate wells:
    i. cDNA sample for classical qPCR condition.
    ii. UltraPure DNase/RNase-free Distilled water for the “w/o cDNA sample” control condition.
  e. Run the qPCR using the standard SYBR Green Amplification protocol (with dissociation curve), selecting the appropriate well position and fluorophore filter and using the written qPCR program (see the programs preparation sub-section in the before you begin section, Figures 3A and 3C; Table 3).

**Table:** 

Reagent	Final concentration	Amount
cDNA sample (containing 1- Random hexamers, 2- dNTP mix, 3- First Strand Buffer, 4- DTT, 5- RNase OUT, 6- SuperScript III RT)	Variable concentration (1– 250 nM, 2– 50 μM, 3– 0.1X, 4– 500 μM, 5– 125 μM, 6– 0.25 U/μL)	1 μL
Forward primer (10 μM)	500 nM	0.5 μL
Reverse primer (10 μM)	500 nM	0.5 μL
i*Taq* Universal SYBR Green Supermix (2X)	1X	5 μL
UltraPure DNase/RNase-free Distilled water	N/A	3 μL
Total	N/A	10 μL

***Note:*** If necessary, the qPCR plate can be briefly centrifuged at 1 000 rpm (95 G) and stirred for 1–2 min.

**CRITICAL:** The primer couples should be adapted to the annealing (hybridization) temperature of 60°C allowing for a separation of the hybridization (60°C) and elongation (72°C) steps.

**CRITICAL:** The cDNA sample could be diluted to avoid using a volume of 1 μL, due to the relative inaccuracy of pipetting this low volume. The volume of water in the master mix C has then to be adapted.

### UV absorbance measurement for a step-by-step monitoring

**Timing: 0.5–1 hour**

This step describes the procedure for the estimation of the RNA concentration of samples in using a spectrophotometer UV and measuring the absorbance of samples at 230, 260, 270 and 280 nm. This step will allow to assess the efficiency of cell lysis, precipitation but also the RNA purification.

40. Make the baseline.

**CRITICAL:** Use the appropriate buffer for baseline, *i.e.*, the G4RP lysis buffer for raw lysate supernatant and UltraPure DNase/RNase-free Distilled Water for purified RNAs. An inappropriate baseline could lead to abnormal UV absorbance curves (e.g., strong negative part of the curve).

**CRITICAL:** Heat the G4RP lysis buffer at 30°C–40°C to allow the SDS being completely dissolved if needed.

41. Measure the UV absorbance between 200-340 nm.

***Note:*** The drop of sample used for the quantification can be take back for the gel electrophoresis.

**CRITICAL:** Clean well the material between each measurement to avoid cross-contamination between samples (e.g., wash with water, then with 70% ethanol and dry it).

**CRITICAL:** For too concentrated samples, favor the use of cap (the 50X instead of 10X) for fiber-optic ultra-micro cells or device possessing adjustable path length instead of diluting the sample which will make the baseline not appropriated anymore (*i.e.*, pH, salt and/or SDS concentration discord).

42. Recover the peak absorbance value at following wavelengths (A_λ_):
  a. 230 nm for organic compounds absorbance.
  b. 260 nm for nucleic acids (DNA and RNA) absorbance.
  c. 270 nm for phenol (TRIzol)/chloroform absorbance.
  d. 280 nm for proteins absorbance.
43. Calculate the following quality and quantity ratios for monitoring your G4RP.v2 protocol:
  a. R1 = A_260 nm_/A_230 nm_ for organic compounds contamination.
    i. If R1 = 1.8–2.2, pure nucleic acids.
    ii. If R1 < 1.8, contamination.
  b. R2 = A_260 nm_/A_270 nm_ for phenol (TRIzol)/chloroform contamination.
    i. If R2 = 1.2, clean nucleic acids.
    ii. If R2 < 1.2, contamination.
  c. R3 = A_260 nm_/A_280 nm_ for proteins contamination.
    i. If R3 ≈ 2, pure RNA.
    ii. If R3 ≈ 1.8, pure DNA.
    iii. If R3 < 1.8, proteins contamination.
  d. Estimated RNA concentration (in ng/μL) = A_260 nm_ ∗ 40.

### Gel electrophoresis for a step-by-step monitoring

**Timing: 2.5–3 h**

This step describes the procedure for the analysis of samples in running them on a denaturing agarose gel. This step will allow to check the efficiency of cell lysis, precipitation but also the RNA purification.

44. Make the denaturing 0.026% (w/v) bleach 1.5% (w/v) agarose gel.

***Note:*** See the materials and equipment section for the recipe and protocol to make the gel.

45. Let the agarose gel submerged in fresh TBE buffer (1X).
46. Prepare sample before loading:
  a. Make the following mixture:
    i. 4 μL of samples (e.g., raw lysate supernatant, purified RNA, others).
    ii. 0.5 μL of the 2X Loading Dye solution (provided with the RiboRuler High Range RNA Ladder product).
  b. Homogenize gently.
  c. Incubate for 10 min at 70°C.
  d. Chill on ice 5–10 min.

***Note:*** This volume of the 2X Loading Dye solution is sufficient to benefit from the heavy glycerol and colored dye properties.

**Pause point:** Store at −20°C for up to a week with the 2X Loading Dye solution (before the incubation).

47. Load cautiously samples on gel wells:
  a. 4 μL for samples.
  b. 2 μL for the RiboRuler High Range RNA Ladder solution (provided with the RiboRuler High Range RNA Ladder product).
48. Run the gel at 100 V for 1.75 h (105 min) at 4°C.

***Note:*** For a 1% (w/v) agarose gel, the bromophenol blue and xylene cyanol dyes migration correspond to a 200–500 and 3 000–4 000 bases nucleic acid, respectively.

**CRITICAL:** The electric tension being the limiting parameter, you can set up the power supply at 100 V (electric tension), 300 mA (electric current) and 30 W (power). During the running, the power will be at ∼12 W.

49. Reveal the gel:
  a. Prepare the revelation solution:
    i. 7.5 μL of SYBR Gold Nucleic Acid Gel Stain (10,000X).
    ii. 75 mL of TBE buffer (1X).
  b. Mix well.
  c. Incubate the gel in this revelation solution for 20 min at room temperature (25°C) on a benchtop laboratory rocker and protected from the light.
  d. Image the gel with your standard gel imager and appropriate SYBR filters measurement (see problem 10, troubleshooting section).

## Expected outcomes

This protocol describes the different steps required to purify and identify RNA G4s from human cancer cells, treated or not with a G4 interacting agent. The G4RP.v2 protocol was applied with 3 treatment conditions (i.e., non-treated cells, BRACO-19- or PhpC-treated cells) and three different TASQs (BioTASQ, BioCyTASQ and biotin-clicked ^az^MultiTASQ *vs.* biotin as control), in order to quantify two different G4s (*NRAS* and *VEGFA*).

**Figure 4:**
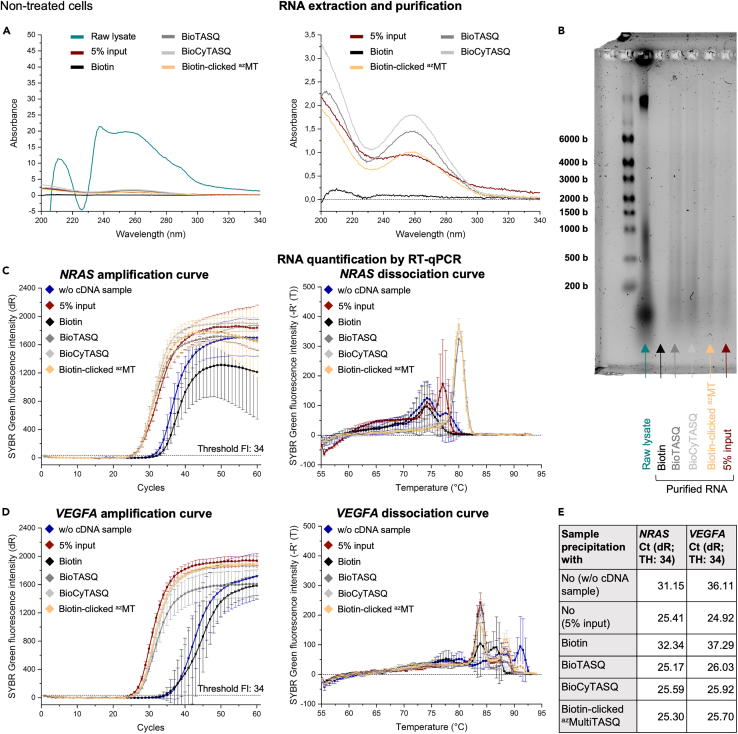
Data obtained through the application of the G4RP.v2 protocol on non-treated MCF7 cells (A) Representative UV absorbance curves of the raw lysate supernatant (i.e., sample obtained after the cell lysis) (green line), the 5% input sample (i.e., RNA purified directly after the cell lysis) (red line), the purified RNA obtained after the control precipitation with biotin (black line) and the purified RNAs obtained after the G4-precipitation with BioTASQ (dark gray line), BioCyTASQ (light gray line) or biotin-clicked ^az^MultiTASQ (yellow line). (Right) Zoom on curves. For the raw lysate and the others samples (purified RNA), dilution factors of 50 and 10 have been applied to Abs values, respectively. (B) Quality check of samples by a 0.026% (w/v) bleach 1.5% (w/v) denaturing agarose gel electrophoresis (with RiboRuler High Range RNA Ladder). (C and D) Representative amplification (left) and dissociation (right) curves obtained after the RT-qPCR analysis of the G4-mRNAs (C) *NRAS* and (D) *VEGFA* from the different samples. UltraPure RNase/DNase-free Distilled water was used instead of purified RNA samples in the “w/o cDNA sample” (dark blue line). Error bars represent SD from the mean for three technical replicates of a representative experiment (among three independent experiments). (E) Summary of Ct (dR) values obtained with the amplification curves (threshold fluorescence intensity = 34) and calculated G4RP-RT-qPCR signal.

**Figure 5:**
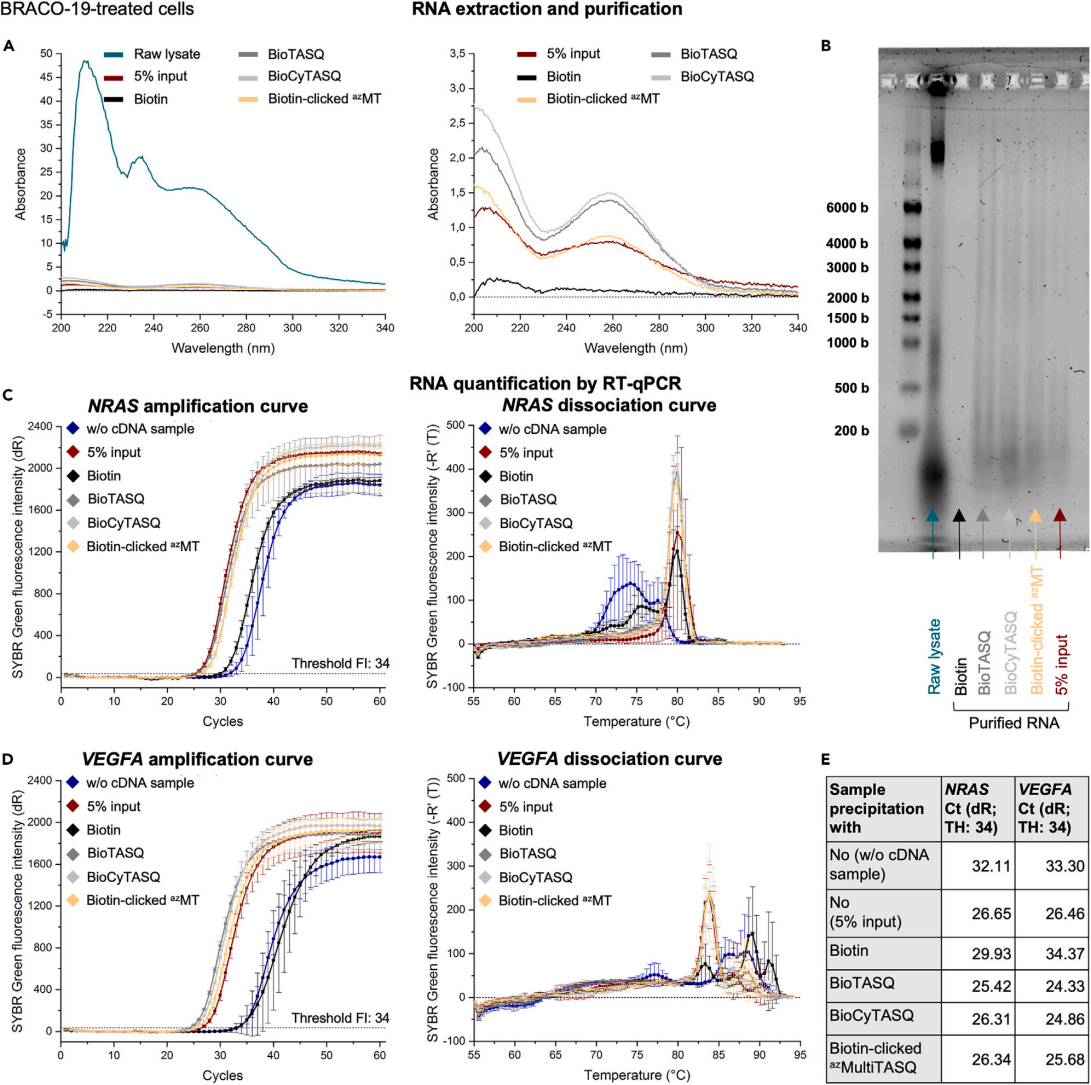
Data obtained through the application of the G4RP.v2 protocol with BRACO-19-treated MCF7 cells (A) Representative UV absorbance curves of the raw lysate supernatant (i.e., sample obtained after the cell lysis) (green line), the 5% input sample (i.e., RNA purified directly after the cell lysis) (red line), the purified RNA obtained after the control precipitation with biotin (black line) and the purified RNAs obtained after the G4-precipitation with BioTASQ (dark gray line), BioCyTASQ (light gray line) or biotin-clicked ^az^MultiTASQ (yellow line). (Right) Zoom on curves. For the raw lysate and the others samples (purified RNA), dilution factors of 50 and 10 have been applied to Abs values, respectively. (B) Quality check of samples by a 0.026% (w/v) bleach 1.5% (w/v) denaturing agarose gel electrophoresis (with RiboRuler High Range RNA Ladder). (C and D) Representative amplification (left) and dissociation (right) curves obtained after the RT-qPCR analysis of the G4-mRNAs (C) *NRAS* and (D) *VEGFA* from the different samples. UltraPure RNase/DNase-free Distilled water was used instead of purified RNA samples in the “w/o cDNA sample” (dark blue line). Error bars represent SD from the mean for three technical replicates of a representative experiment (among three independent experiments). (E) Summary of Ct (dR) values obtained with the amplification curves (threshold fluorescence intensity = 34) and calculated G4RP-RT-qPCR signal.

**Figure 6:**
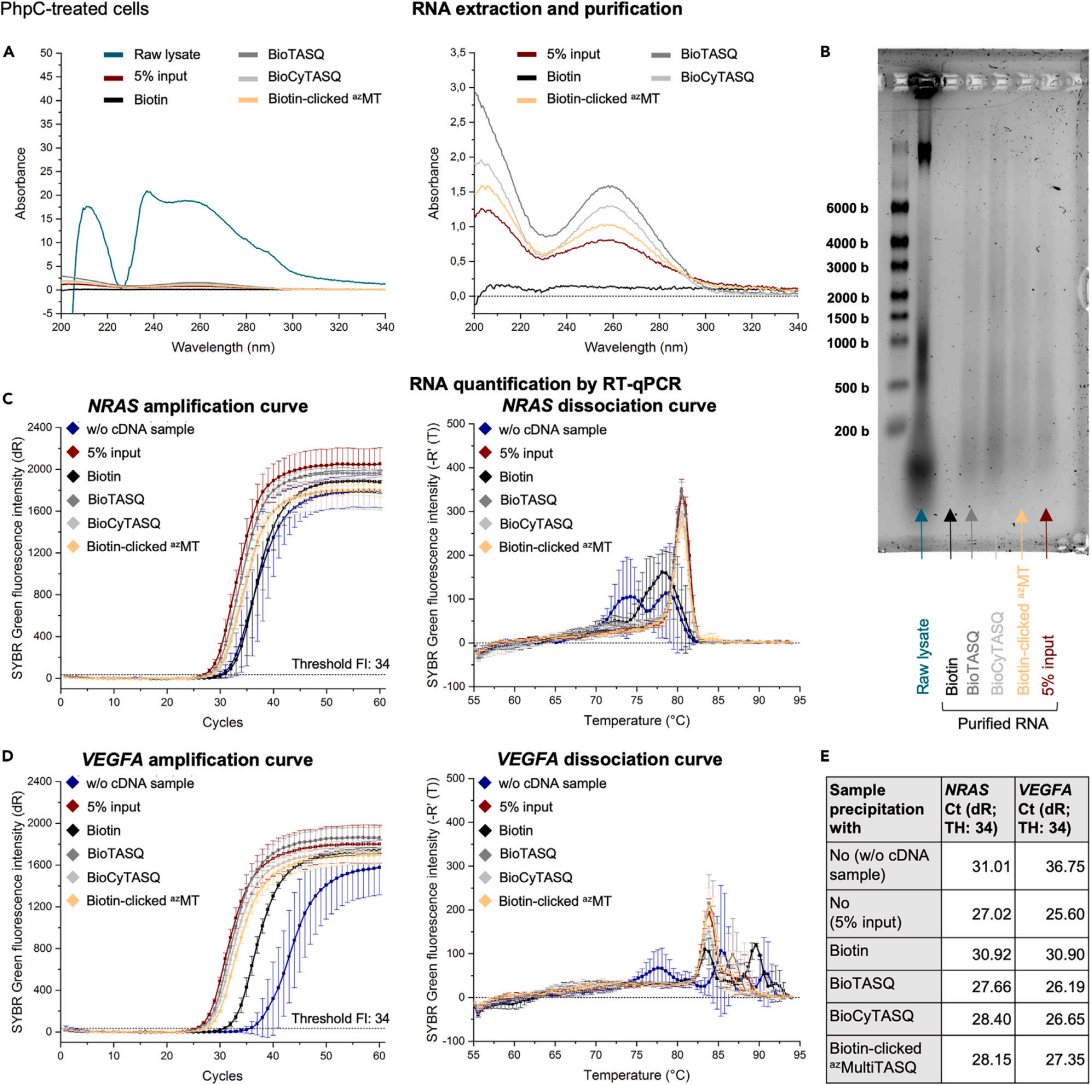
Data obtained through the application of the G4RP.v2 protocol with PhpC-treated MCF7 cells (A) Representative UV absorbance curves of the raw lysate supernatant (i.e., sample obtained after the cell lysis) (green line), the 5% input sample (i.e., RNA purified directly after the cell lysis) (red line), the purified RNA obtained after the control precipitation with biotin (black line) and the purified RNAs obtained after the G4-precipitation with BioTASQ (dark gray line), BioCyTASQ (light gray line) or biotin-clicked ^az^MultiTASQ (yellow line). (Right) Zoom on curves. For the raw lysate and the others samples (purified RNA), dilution factors of 50 and 10 have been applied to Abs values, respectively. (B) Quality check of samples by a 0.026% (w/v) bleach 1.5% (w/v) denaturing agarose gel electrophoresis (with RiboRuler High Range RNA Ladder). (C and D) Representative amplification (left) and dissociation (right) curves obtained after the RT-qPCR analysis of the G4-mRNAs (C) *NRAS* and (D) *VEGFA* from the different samples. UltraPure RNase/DNase-free Distilled water was used instead of purified RNA samples in the “w/o cDNA sample” (dark blue line). Error bars represent SD from the mean for three technical replicates of a representative experiment (among three independent experiments). (E) Summary of Ct (dR) values obtained with the amplification curves (threshold fluorescence intensity = 34) and calculated G4RP-RT-qPCR signal.

The amount of recovered RNA depends on the conditions (control *vs.* G4RP) but also on the selected TASQ; this is now monitored by UV absorbance and gel electrophoresis (Figures 4A, 4B, 5A, 5B, 6A, and 6B). The value of UV absorbance at 260 nm is used for estimating the RNA concentration (in ng/μL).

For the raw lysate supernatant from *ca.* 20 × 10^6^ cells, we obtained *ca*. 380 μL of samples which strongly absorb UV light (Figures 4A, 5A, and 6A, green line), leading to an estimated nucleic acids (DNA and RNA) concentration of 736–856 ng/μL, that is, 280–325 μg of nucleic acids. The gel electrophoresis was performed with 4 μL of these samples (*i.e*., 2.9–3.4 μg), which leads to intense bands (the high band for DNA, the middle and low bands for RNA, Figures 4B, 5B, and 6B, green arrow). Of note, the DNA high band disappears after RNA purification and is absent when the cell lysis is performed with a douncer which is supposed to let *nuclei* intact (see the resource availability section).

After the precipitation and purification steps, the amount of purified RNA varies: with the biotin control, 30 μL of purified RNA were obtained with almost no UV absorbance (Figures 4A, 5A, and 6A, black line), leading to an estimated RNA concentration of 3.2–5.6 ng/μL (*i.e*., 0.1–0.2 μg). By gel electrophoresis, these samples (loaded amount = 12.8–22.4 ng) led to empty lanes (Figures 4B, 5B, and 6B, black arrow). Conversely, with TASQ tools, 30 μL of purified G4-RNA were obtained with well-defined UV absorbance curves (Figures 4A, 5A, and 6A, dark gray, light gray and yellow lines for BioTASQ, BioCyTASQ and biotin-clicked ^az^MultiTASQ, respectively), corresponding to an estimated RNA concentration of 35.2–71.2 ng/μL (*i.e*., 1.6–2.1 μg). This >10-fold difference highlights the efficiency of the TASQ tools to purify RNA from cell lysate. Gel electrophoresis (Figures 4B, 5B, and 6B) confirms these results (loaded amount = 140.8–284.8 ng). Globally, the G4-specific tools BioTASQ and BioCyTASQ seem to allow for a better recovery of RNA (Figures 4A, 4B, 5A, 5B, 6A, and 6B, dark and light gray lines/arrows respectively).

The quantification of *NRAS* and *VEGFA* G4s by RT-qPCR showed amplification curves with a similar pattern: Ct (dR) values > 30 for the two controls (i.e., w/o cDNA and biotin-precipitated samples) and Ct (dR) values < 30 for the G4RP conditions (i.e., G4-precipitation with BioTASQ, BioCyTASQ or biotin-clicked ^az^MultiTASQ) (Figures 4C, 4D, 4E, 5C, 5D, 5E, 6C, 6D, and 6E). These results indicate that no significant *NRAS* or *VEGFA* G4 amplification occurred in control samples (blue and black lines), while the TASQ tools (dark gray, light gray and yellow lines) allow for an efficient extraction of these two G4s, with Ct (dR) values between 25.17 and 26.03.

The dissociation curves obtained for *NRAS* G4 (Figures 4C, 5C, and 6C) are a good example of homogenous PCR products (dark gray, light gray and yellow lines for BioTASQ, BioCyTAQ and biotin-clicked ^az^MultiTASQ, respectively) as they display an unique dissociation peak (around 80°C).

Of note, optimization experiments of the G4RP.v2 protocol (see the resource availability section) have been also conducted with 12 × 10^6^ MCF7 cells allowing for obtaining *ca*. 177.7 μg (*ca.* 380 μL at 467.6 ng/μL) of extracted RNA (before G4-precipitation and RNA purification) and then 0.9 μg (*ca.* 30 μL at 29.0 ng/μL) of purified G4-RNA (with the BioCyTASQ). This amount of RNA, which respects the recommendation of the SuperScript III Reverse Transcriptase provider of 10 pg - 5 μg of total RNA for reverse transcription (RT) reaction, was sufficient to obtain a correct RT-qPCR signal for *NRAS* mRNA. With this in mind, starting with 10 × 10^6^ MCF7 cells could be suited to the G4RP.v2 protocol.

For the final data representation and discussion about the influence of the G4-interacting compounds (BRACO-19 or PhpC) on the folding of the *NRAS* G4 assessed by the G4RP.v2 protocol, see the resource availability section.

## Quantification and statistical analysis

For the analysis of the qPCR data, we used the same formula than in the original study of Yang et al.^23^ for comparison purpose (the analysis pipeline is detailed below). The classical qPCR formula 2^−ΔΔCt^ can be used, however, care must be taken to ensure that the appropriate experimental conditions have been implemented (e.g., RT-qPCR quantification of a reference RNA target).

The analysis of the qPCR data is performed according to the following steps: *i-* set the threshold for the SYBR Green fluorescence intensity (FI), *ii-* recover the Ct (dR) values, *iii-* normalize the Ct (dR) values using the G4RP-RT-qPCR signal formula, as follows.

1. Set the FI threshold:
  a. The thermocycler (Agilent) displays the amplification curves on a y = f(x)-type graph with the following parameters: baseline-corrected raw SYBR Green FI (dR) on cycles number (cycles).
  b. Put the dR in log(y).
  c. Focus only in the almost-vertical and parallel part of exponential curves and select the lowest FI validating these two last prerequisites (= the inflection point) to place the threshold horizontal line.
2. Recover the Ct (dR) values:
  a. Put the dR back in linear y.
  b. Take the cycle number corresponding to the intersection point between the previous threshold FI horizontal line and the appropriate amplification curve corresponding to an experimental condition = this is the Ct (dR) value of your experimental condition.
3. Normalize the Ct (dR) values:
  a. Calculate the “Mean Ct input” which is the mean of Ct (dR) values for the 5% input control (biological triplicate).
  b. Calculate the G4RP-RT-qPCR signal = 5∗(2^(Mean Ct input) – Ct G4RP or biotin^), with each technical Ct (dR) of G4RP (= G4-precipitation with TASQ) or biotin experimental condition.
  c. For each precipitation and target condition (e.g., *NRAS* G4 precipitation with BioCyTASQ, *VEGFA* G4 precipitation with biotin control):
    i. Calculate the technical mean G4RP-RT-qPCR signal for each biological replicate (with the technical replicates).
    ii. Calculate the mean of G4RP-RT-qPCR signal (with the biological replicates).
  d. Make your data representation with these mean normalized values.
  e. Analyze your data (see problems 7, 8, and 9, troubleshooting section).

## Limitations

The G4RP.v2 protocol, as the original G4RP protocol, is a user-friendly protocol which however requires several attempts to be fully mastered. The monitoring steps (UV measurement and gel electrophoresis) are critical to assess the efficiency of every step of the protocol, unblinding the protocol advance and comforting the manipulator during the experiments. These monitoring steps are thus useful for optimization and/or adaptation of the protocol to a new environment and/or purpose.

Nonetheless, this protocol has conceptual limitations: if the purpose of the study is to assess the effect of a G4-interacting agent treatment on the abundance of a G4-RNA, the ability of the molecule to enter cells, its possible chemical alteration in biological media, and the accessibility of its target can affect the outcomes of the experiments.

Another limitation is sensitivity. The protocol may not succeed if the G4 target has low abundance or is too stable. Indeed, the presence of a G4 structure in the purified RNA and then in the cDNA generated by RT could be a problem for polymerase processing (e.g., SuperScript III reverse transcriptase and i*Taq* polymerase) during the RT-qPCR steps. It can possibly lead to a low efficiency of the polymerase-dependent steps, and even to a total inhibition of the polymerase activity if the G4s are too stable.

Finally, several technical limitations of the G4RP.v2 protocol can be mentioned: the quantity and quality of the biological material are critical, meaning that *i-* the number of cells is important, *ii-* the crosslinking step has to be fast to limit cell component degradation, *iii-* the buffers and solutions must be prepared in RNase-free water (e.g., ddH_2_O or DEPC-treated water) and a sterile environment (e.g., under a standard biosafety cabinet, with 0.2-μm filtration, etc.), and *iv-* the manipulation must be done on ice or at 4°C for most of the steps, to preserve the integrity of RNA and maximize its recovery. If the used TASQ necessitates a click reaction for the introduction of a biotin handle, access to an HPLC-MS device is mandatory to ascertain the efficiency of this reaction. Also, the type and use of commercial kits can influence the corresponding steps (e.g., RNA purification or RT-qPCR). Also, the timing is important: for instance, the gel electrophoresis analysis works well only if the samples are loaded and ran on the day of extraction (and not the following day, day 5).

## Troubleshooting

### Problem 1

Cell death due to G4-interacting agent toxicity (e.g., floating cells, reflective condensates inside the cells, cell density lower than usual) (see step 2-a, step-by-step method details section).

### Potential solution

• Calculate the IC_50_ value for every used G4-interacting agent to adapt its concentration.
• Decrease the G4-interacting agent concentration, whenever possible.

### Problem 2

There are too few cells for the crosslinking and lysis steps (see step 4-c, step-by-step method details section).

### Potential solution

• Check or calculate the doubling time of used cell lines to be sure that enough cells were seeded.
• Use several cell density conditions and count the cell number after a night and 48 h (to mimic the incubation time in the G4RP.v2 protocol).
• Check if cells are adherent after seeding and after one night. If not, the addition of the G4-interacting agent can affect the cell capacity to adhere and then, cause their death indirectly.

### Problem 3

Issues during the lysis with a needle-equipped syringe (the cell suspension in G4RP lysis buffer and/or bubbles pass through the gasket, loss of cells/lysate) (see step 9, step-by-step method details section).

### Potential solution

Disassemble gently the syringe, aspirate the buffer/bubble with a pipette, put it back in the microtube and reassemble the syringe.

### Problem 4

Issue to recover only the transparent RNA-containing top aqueous phase (see step 33, step-by-step method details section).

### Potential solution

• Take delicately the major part of this phase and let a security volume of it in order to avoid pipetting the middle white-colored interphase (or the bottom pink-colored organic phase).
• Try Phasemaker-type microtubes containing a polymer able to separate physically the aqueous and organic phases (not successfully attempted in our hands)

### Problem 5

The quantity of purified RNA is not satisfying (see step 36-b, step-by-step method details section).

### Potential solution

• Cell density: increase the number of cells to be seeded (step 1).
• Material degradation: use only RNase-free water, work only in a sterile environment, on ice or at 4°C for most of the steps, keep some materials for some hours at 4 or -20°C (e.g., needle, cell scraper), avoid talking in front of the working area (or wear a protective mask).
• Crosslinking: using formaldehyde is mandatory to preserve G4 structures but a too high formaldehyde concentration makes the reverse crosslinking step difficult (step 5-d).
• Lysis: the number of pipetting can be increased and do not hesitate to force moderately during the expulsion of cells suspension in G4RP lysis buffer (step 9). Change or combine different lysis methods such as chemical (e.g., buffer with detergent; try harder lysis buffer if necessary), mechanical (e.g., with a needle-equipped syringe, cell scraper or douncer) or physical (e.g., sonication) methods:
  ○ With a cell scraper: seed cells in Petri dish, proceed according to the G4RP.v2 protocol until the step 3, then wash the adherent cells with DEPC-PBS once, add the Formaldehyde (1% w/v)/Fixing buffer (1X), incubate and quench with glycine, incubate and wash with DEPC-PBS once, add the G4RP lysis buffer, scrape cells vigorously and transfer the suspension into a 1.5 mL microtube. The subsequent steps are then performed according to the G4RP.v2 protocol.
  ○ With a douncer: seed cells in flask or Petri dish, proceed according to the G4RP.v2 protocol until the step 8, then put the cell suspension at the bottom of the cylindrical mortar, make 50 dounces with the tight pestle and transfer the suspension into a 1.5 mL microtube. The subsequent steps are then performed according to the G4RP.v2 protocol. Care must be taken to ensure that the douncer is well-cleaned between experiments to avoid cross-contaminations.
• Amount of raw lysate supernatant used for the G4-precipitation (with TASQ and beads): make several attempts with higher volumes. (step 12-a).
• G4 precipitation: increase the amount of used TASQ or beads, or the duration of the incubation (steps 17–19). However, increasing these parameters will also increase the nonspecific binding.
• Reverse crosslinking step: increase the duration of this heating (70°C) step to free more material but care must be taken as a too long heating of RNA samples can affect their stability (step 26-b).
• Binding capacity of column used for RNA purification: samples can be divided in two Zymo-Spin IC Columns (binding capacity = 10 μg) and then gathered after purification or try other column with a higher binding capacity as the Zymo-Spin IIC Columns (binding capacity = 50 μg) (step 35-b).
• Do not hesitate to keep supplementary samples (e.g., unbound material samples after the precipitation, washing supernatants after the washings) to analyze them by the two monitoring methods (i.e., UV measurement and gel electrophoresis) to detect limitations or problems (steps 21-b and 22-a-b).

### Problem 6

The quality of purified RNA recovered after purification is not satisfying (see the UV absorbance measurement step in Step-by-step section) (see step 36-b, step-by-step method details section).

### Potential solution

• Perform a second in-column RNA purification (step 35).
• Quality checks might not be reliable for samples with a too low concentration or depending on the sensitivity of the device used for the estimation of this concentration. Increasing the starting material amount can address this issue.

### Problem 7

The quantification by RT-qPCR does not work (Ct (dR) values > 30–35 cycles) (see the quantification and statistical analysis section).

### Potential solution

• Low expression of the RNA target in cells: check this in working with the totality of the input (= no precipitation) and make a RT-qPCR quantification with this highly RNA-rich sample (step 12-a).
• Primers used for qPCR: check if the two primers are not too distant from each other (*ca.* 200 bases) and if their annealing temperature is compatible with the 60°C of the hybridization step (see Figure 3).
• RT reaction: try a more specific method using, for instance, polyT primers for mRNA RT to cDNA (step 37-a).
• RNA quantity: increase the concentration of purified RNA used for the RT step (step 37-c) (see problem 6).
• cDNA quantity: increase the volume of cDNA used in the qPCR reaction (step 39-d).

### Problem 8

The quantification by RT-qPCR is nonspecific/homogenous (e.g., two exponential-phases amplification curves, many peaks in dissociation curves) (see the quantification and statistical analysis section).

### Potential solution

• The presence of DNA or nonspecific RNA sequences (i.e., not G4) in the purified RNA samples: improve the quality of the G4-precipitation step adding supplementary washing steps (with G4RP wash buffer and/or DEPC-PBS) (step 22) and/or a pre-clearing step before precipitation: incubate the fraction of raw lysate supernatant with beads alone, then magnet the beads and keep only the supernatant of this pre-cleared raw lysate to remove the components which interact nonspecifically with beads. Then proceed to classical precipitation with the pre-cleared raw lysate plus other beads and a G4-specific TASQ tool (or biotin). It can also be good to improve the efficiency of the RNA purification (see problems 6 and 7).
• Primers: they might be not specific enough and/or generate primer-dimers. Redesign them.
• Contamination of the qPCR reaction: check the contamination/purity of the water used and/or change for a suitable qPCR water, using a clean material in a dedicated area during the qPCR plate preparation (if possible, the areas of qPCR plate preparation and plate reopening after the qPCR must be separated).
• Reagents: avoid freeze/thaw cycles, notably for the i*Taq* Universal SYBR Green Supermix (2X).

### Problem 9

The G4-interacting agent treatment does not have an effect on the RNA target abundance and/or expression (see the quantification and statistical analysis section).

### Potential solution

• Use several concentrations of G4-interacting agent (step 2-a).
• If possible, check the ability of used G4-interacting agent to enter cells.

### Problem 10

The nature of gel electrophoretic bands is unclear (see step 49-d, step-by-step method details section).

### Potential solution

• Lyse cells using a douncer to observe the RNA bands in absence of the DNA band (step 9) (see problem 5). A band with a too high apparent molecular weight can correspond to samples not properly lysed.
• Increase the duration of the heating (70°C) step of reverse crosslinking to free more material but care must be taken as a too long heating of RNA samples can affect their stability (step 26-b).
• Make an enzymatic digestion with DNase I or RNase H.

### Problem 11

The length of the G4RP.v2 protocol *per se* and the organization of the working days.

### Potential solution

Make necessary arrangements in accordance with these long days of manipulation.

## Resource availability

### Lead contact

Further information and requests for resources and reagents should be directed to and will be fulfilled by the lead contact, David Monchaud (david.monchaud@u-bourgogne.fr).

### Technical contact

Questions about the technical details concerning the protocol should be directed to the technical contact, Jérémie Mitteaux (jeremie.mitteaux@u-bourgogne.fr).

### Materials availability

• BioCyTASQ used in this study is commercially available via Merck Millipore.^47^
• The other molecular tools used in this study (BioTASQ and ^az^MultiTASQ) are available from the lead contact without restriction.
• The PhpC compound used in this study was kindly provided by Robert H. E. Hudson (rhhudson@uwo.ca) from the Department of Chemistry of The University of Western Ontario (London, N6A 5B7 ON, Canada).

### Data and code availability

• Original G4RP-RT-qPCR data have been published.^1^
• The datasets generated during this study are available at These: 2023UBFCK097^41^ and FigShare: 25957162.^42^

## Acknowledgments

This work was supported by the CNRS, iSITE BFC (COMUE UBFC, PIA2, grant no. UB21018.MUB.IS), the Agence Nationale de la Recherche (ANR-17-CE17-001001, DEMENTIA), and the European Union (PO FEDER FSE Bourgogne 2014/2020 programs, grant no. BG0021532, SEQUENTIA). The authors are grateful to Zi Wang and Prof. Robert H. E. Hudson (UWO, Canada) for providing PhpC and to Sandy Raevens and Dr. Ibai E. Valverde (ICMUB, Dijon, France) for preparing TASQ tools.

## Author contributions

Conceptualization, D.M.; methodology, J.M.; software, J.M.; validation, J.M.; formal analysis, J.M.; investigation, J.M.; resources, D.M.; data curation, J.M. and D.M.; writing – original draft, J.M.; writing – review and editing, J.M. and D.M.; visualization, J.M.; supervision, D.M.; project administration, D.M.; funding acquisition, D.M.

## Declaration of interests

BioCyTASQ is commercialized by Merck KGaA; ^az^MultiTASQ is a patented product (CNRS & Univ. Bourgogne, international patent WO2021198239 (A1) 2021/10/07).
